# Non-invasive real time monitoring of yeast volatilome by PTR-ToF-MS

**DOI:** 10.1007/s11306-017-1259-y

**Published:** 2017-08-31

**Authors:** Iuliia Khomenko, Irene Stefanini, Luca Cappellin, Valentina Cappelletti, Pietro Franceschi, Duccio Cavalieri, Tilmann D. Märk, Franco Biasioli

**Affiliations:** 1 Research and Innovation Centre, Fondazione Edmund Mach, Via E. Mach 1, San Michele all’Adige, TN Italy; 2 Institute for Ion Physics and Applied Physics, University of Innsbruck, Technikerstr. 25, Innsbruck, Austria; 3 Division of Biomedical Cell Biology, Warwick Medical School, University of Warwick, Coventry, CV4 7AJ UK; 4 Department of Biology, Institute of Biochemistry, ETH Zurich, 8093 Zurich, Switzerland; 5 Biology Department, University of Florence, Via Madonna del Piano 6, Sesto Fiorentino, FI Italy

**Keywords:** *Saccharomyces cerevisiae*, Fermentation, VOCs, Secondary metabolites, Profiling, Direct injection

## Abstract

**Introduction:**

Producing a wide range of volatile secondary metabolites *Saccharomyces cerevisiae* influences wine, beer, and bread sensory quality and hence selection of strains based on their volatilome becomes pivotal. A rapid on-line method for volatilome assessing of strains growing on standard solid media is still missing.

**Objectives:**

Methodologically, the aim of this study was to demonstrate the automatic, real-time, direct, and non-invasive monitoring of yeast volatilome in order to rapidly produce a robust large data set encompassing measurements relative to many strains, replicates and time points. The fundamental scope was to differentiate volatilomes of genetically similar strains of oenological relevance during the whole growing process.

**Method:**

Six different *S. cerevisiae* strains (four meiotic segregants of a natural strain and two laboratory strains) inoculated onto a solid medium have been monitored on-line by Proton Transfer Reaction—Time-of-Flight—Mass Spectrometry for 11 days every 4 h (3540 time points). FastGC PTR-ToF-MS was performed during the stationary phase on the 5th day.

**Results:**

More than 300 peaks have been extracted from the average spectra associated to each time point, 70 have been tentatively identified. Univariate and multivariate analyses have been performed on the data matrix (3640 measurements × 70 peaks) highlighting the volatilome evolution and strain-specific features. Laboratory strains with opposite mating type, and meiotic segregants of the same natural strain showed significantly different profiles.

**Conclusions:**

The described set-up allows the on-line high-throughput screening of yeast volatilome of *S. cerevisiae* strains and the identification of strain specific features and new metabolic pathways, discriminating also genetically similar strains, thus revealing a novel method for strain phenotyping, identification, and quality control.

**Electronic supplementary material:**

The online version of this article (doi:10.1007/s11306-017-1259-y) contains supplementary material, which is available to authorized users.

## Introduction

*Saccharomyces cerevisiae* has been exploited since more than 9000 years in food and beverage production (Cavalieri et al. 2003). Different strains have been selected and used as inoculum to conduct alcoholic fermentation, in particular in winemaking, and prevail over other natural microbiota present in fresh must, which could negatively affect the process (Muller-Thurgau 1896). The principal characteristics that a *S. cerevisiae* strain has to bear to be selected for winemaking purposes are: (i) good fitness in fermentative conditions—namely, the ability to metabolize grape must, survive to low pH and temperatures and high concentrations of ethanol, (ii) ability to overgrow the resident fungal and bacterial populations, (iii) good metabolic features (Pretorius 2000). The last characteristic is particularly important for winemaking purposes, since the vast part of the wine flavour is defined by yeast metabolism (Schreier 1979). Indeed, beside ethanol, the concentration and type of higher alcohols, aldehydes, ketones, acetates, esters, and fatty acids, produced by different yeast species and strains have been shown to strongly affect the quality and characteristics of the final product (Herraiz et al. 1989, 1990). The first aim of inoculating grape musts with selected *S. cerevisiae* strains was to reduce the risk of spoilage. Nowadays, however, this feature seems to be no longer sufficient for winemaker intents: the identification of strains able to enhance the wine organoleptic characteristics (i.e. the bouquet of aromatic compounds) is indeed the actual quest (Lambrechts and Pretorius 2000; Pretorius 2000).

So far, the potential of *S. cerevisiae* strains in enhancing the wine aromatic bouquet has been generally assessed by measuring the volatile organic compounds present at the end of alcoholic fermentation (Alves et al. 2015; Romano et al. 2003). Nevertheless, a recent study on the time resolved metabolome of a Chardonnay wine fermentation showed that the metabolome varies considerably during time, suggesting that the yeast metabolism is tightly coupled to the fermentation progress (Richter et al. 2015). It can be expected that the variability of the metabolome could have an even higher extent in different fermentation conditions, considering that the substrate is subjected to modifications induced by a dynamic microbial population. As a consequence, for an appropriate and realistic evaluation of the *S. cerevisiae* strains potential in enhancing the wine aromatic blend, it is fundamental to follow the production of the volatile compounds during the entire process. Although the need for time-resolved metabolic measurements has been acknowledged, the commonly used experimental setups are still technically, time and manpower demanding. In addition, any intervention to the growth conditions or post-sampling matrix processing (i.e. quenching or extraction procedure) can introduce errors affecting the reproducibility of the analysis (Kawase et al. 2014). Recently, methods for the real-time measurement of metabolites have been developed to quantify the volatile organic compounds (VOCs) released from the liquid medium during microbial growth (Link et al. 2015; Mouret et al. 2015). Nevertheless, in such conditions the real VOCs amount effectively produced by the microorganisms should be inferred from the VOCs released from the medium into the air.

Gas chromatography based methods are the reference for the analysis of yeast volatile secondary metabolites (Lloyd et al. 2015). Even if it is able to identify VOCs in low concentrations, this type of analytical technique is inherently slow due to the presence of, at least, one separation stage and this makes it unsuitable for characterizing the dynamic processes happening during yeast growth in real time (Alves et al. 2015). As recently shown in the case of aromatic bakery yeasts (Capozzi et al. 2016; Makhoul et al. 2014) Proton Transfer Reaction—Mass Spectrometry (PTR-MS) coupled to a Time-of-Flight detector (ToF) and a multipurpose sampler was successfully applied for the characterization of yeast VOC profiles for the first 16–24 h of bread leavening in nondestructive and rapid way. This setup also guaranteed temperature stability and automatization of experiments. In the present study, a similar approach made it possible to study VOC release during yeast colony development in real time for longer time for the first time.

In this work, we present a method for the in vivo real time assessment of the *S. cerevisiae* volatilome. We evaluated the approach comparing VOC profiles of two laboratory strains and four meiotic segregants of a natural strain isolated from wine must fermentation (Cavalieri et al. 2000). The new approach showed high reproducibility among different biological replicates and allowed to follow the dynamics of both known and unknown volatile compounds during yeast colony development. In general, we show that the real-time measurement of metabolites produced during yeast growth onto solid medium is cost- and time-effective and allows a characterization of volatile compounds produced by growing microbes.

## Materials and methods

### Yeast culture preparation, growth curve comparison and growth conditions

The yeast strains used in this study are the two laboratory strains BY4741 (Mat a his3Δ1 leu2Δ0 met15Δ0 ura3Δ0) and BY4742 (Mat α his3Δ1 leu2Δ0 met15Δ0 ura3Δ0) and four meiotic segregants of a *S. cerevisiae* strain isolated from Montalcino grapes fermentation (Cavalieri et al. 2000). The genetic variation between the former strains is known to be miniscule (Song et al. 2015). The latter strains share 99.99% genes. The genetic difference between M28 and the two laboratory strains is much larger.

Before the measurement of yeast VOC, the growth curves of the tested strains were compared to acknowledge eventual fitness changes. For this comparison overnight pre-grown strains were inoculated in liquid YPD (1% yeast extract, 2% peptone, 2% dextrose) in 96-well plates and their growth was monitored by measuring the optical density at 600 nm (OD600) for 24 h, a time interval sufficient to highlight the differences in growth ability of the strains (prior reaching the stationary phase). After an overnight pre-culture at 28 °C in liquid YPD the strains were inoculated onto 2 mL solid YPD medium (YPD supplemented with 2% agar) inside a 20 mL vial and closed with a screw cap with a silicon/PTFE septum. A different trend of growth curves was observable among the laboratory strains (BY4741 and BY4742) and the four M28 meiotic derivatives, with the laboratory strains showing a shorter lag phase compared to M28 strains (Table 1). This behaviour is not surprising, since the initial lag is necessary to the yeast to adapt to the environment (the fresh medium) and BY strains have been generated to show good performances in laboratory conditions (Table 1).

**Table 1:** Growth characteristics of the tested strains

	End of lag phase (h)	Doubling time (average hours ± standard deviation)	End of exponential phase (h)	Stationary phase (OD_600_)
BY4741	9	3.39 ± 0.20	15	0.2
BY4742	9	3.56 ± 0.12	15	0.2
M28-1A	13	3.20 ± 0.07	22	0.65
M28-1B	13	4.92 ± 0.58	>24	>0.1
M28-1C	13	3.90 ± 0.18	24	>0.65
M28-1D	13	3.63 ± 0.08	>24	>0.65

Growth curves were inspected in liquid YPD after an overnight pre-culture in the same conditions. Culture growth was evaluated by mean of optical density (OD) measurement at 600 nm

### Headspace PTR-ToF-MS measurements

VOCs produced during yeast growth were measured every 4 h by direct injection of the headspace mixture into a commercial PTR-ToF-MS 8000 apparatus (Ionicon Analytik GmbH, Innsbruck, Austria). The instrumental conditions in the drift tube were as follows: drift voltage 557 V, drift temperature 110 °C, drift pressure 2.30 mbar, affording an E/N value of about 140 Townsend (1 Td = 10^−17^ cm^2^/V.s), where E corresponds to the electric field strength and N to the gas number density. The sampling time per channel of ToF acquisition was 0.1 ns, amounting to 350,000 channels for a mass spectrum ranging up to m/z = 400. Every single spectrum is the sum of 28.600 acquisitions lasting 35 μs each, resulting in a time resolution of 1 s. Sample measurement was performed in 30 cycles resulting in an analysis time of 30 s/sample. Between two measurements 1 min interval was kept to avoid memory effects. The experimental design consisted of 72 yeast samples (6 different strains × 12 replicates), 12 vials with YPD solid medium only and six vials with air of the laboratory where yeast inoculations were performed. Measurements were performed in an automated way by using a multipurpose GC automatic sampler (Autosampler, Gerstel GmbH, Mulheim am Ruhr, Germany) as described in Makhoul et al. 2014. Each sample was measured every 4 h which was enough for VOC accumulation in the headspace of the vial according to the preliminary trials. A gas calibration unit (GCU, Ionicon Analytik GmbH, Innsbruck, Austria) was employed to generate zero air for flushing sample headspace in order to prevent the microbiological and chemical contamination of a sample headspace.

Due to high ethanol concentration in the sample headspace, in order to prevent primary ion depletion and ethanol cluster formation (Romano et al. 2014) an Argon dilution system was applied after headspace sampling. The dilution ratio was 1 part of headspace to 3 parts of Argon. The Argon flow rate was 120 sccm and was controlled by a multigas controller (MKS Instruments, Inc). The total flow rate of the instrument was set to 160 sccm. Supplementary Fig. 1 shows schematically the experimental setup used for headspace measurements.

### FastGC PTR-ToF-MS

Due to the high mass resolution provided by the TOF analyzer it is possible to separate and tentatively identify isobaric compounds. However, with this technique it is impossible to distinguish isomeric compounds. Moreover, as already mentioned, direct headspace air injection into the PTR-ToF-MS is problematic in the case of samples with high ethanol concentration in a headspace. To overcome both problems the PTR-ToF-MS was also coupled with a fastGC add-on (Ionicon Analytik GmbH, Innsbruck, Austria) as it is described elsewhere (Romano et al. 2014). The schematic setup is presented in Supplementary Fig. 2. The polar capillary column [MXT^®^-WAX (Siltek^®^ - treated stainless steel), 6 m, 0.25 mm ID, 0.25 μm df] was maintained under pure N_2_ with a constant flow rate of 4 sccm. Sample headspace air was injected with the flow rate of 150 sccm into a fastGC sampling loop (Supplementary Fig. 2) for 4 s, guaranteeing its total filling. The chromatographic measurement was registered for 130 s with the thermal ramp from 40 to 220 °C which resulted the thermal gradient of 2.25 °C/s (Supplementary Fig. 3). Between two measurements an interval of 100 s was set to prevent memory effects.

The acquisition time was decreased in order to obtain a higher (retention) time resolution in the chromatograms. Each spectrum consisted of 1000 acquisitions lasting 35 μs each, resulting in a time resolution of 210 ms.

One round of measurements by means of fastGC PTR-ToF-MS was performed during the stationary phase on the fifth day of the experiment.

### Data processing and statistical analysis

Data processing of PTR-ToF-MS spectra with or without fastGC included dead time correction, external calibration and peak extraction steps performed according to a procedure described elsewhere (Cappellin et al. 2010). The baseline of the mass spectra was removed after averaging the whole measurement and peak detection and peak area extraction was performed by using a modified Gaussian to fit the data (Cappellin et al. 2011). To determine the concentrations of volatile compounds in ppbv (part per billion by volume) the formulas described by Lindinger et al. were used assuming a constant reaction rate coefficient (k_R_ = 2 × 10^−9^ cm^3^/s) for H_3_O^+^ as primary ion (Lindinger et al. 1998). For H_3_O^+^ as a primary ion, this introduces a systematic error for the absolute concentration for each compound that is in most cases below 30% and can be accounted for if the actual rate constant is available (Cappellin et al. 2012).

Total mass spectrum of yeast, medium and blank samples measured by PTR-ToF-MS contained more than 300 peaks. Two-way ANOVA (strain and time, p < 0.01 with Bonferroni correction) was applied to select mass peaks of yeast samples significantly different from blank ones. Mass peak belonging to ^13^C, ^18^O, and ^27^S isotopologues, water and ethanol clusters were excluded from the dataset. This procedure reduced the dataset to 70 mass peaks. When a monoisotopic mass peak was saturated its isotopologue was taken into account. The data related to two samples of BY4741 and one of BY4742 were eliminated because of evident anomalies probably related to environmental contaminations.

Relative standard deviation (RSD = standard deviation/mean × 100%) was selected as an approach for characterizing measurement variability (Parsons et al. 2008). It was calculated for each selected mass peak for each cycle of measurements.

Principal component analysis (PCA) was performed on the log transformed and mean centered data. 50 out of 70 mass peaks of this dataset were assigned a sum formula and 34 of these formulas were tentatively identified as one or more compounds based on literature and GC-MS measurement of one sample of each yeast strain at the end of the experiment (Supplementary Table I). The tentatively identified compounds belong to different chemical classes such as alcohols, aldehydes, esters, organic acids, ketones, and sulfur compounds. For a better visualization, curves of all mass peaks were smoothed by fitting a cubic spline with a smoothing parameter equal to 0.5. Since samples cannot be measured at exactly the same time, values at all time points were predicted based on this fitting.

In the case of fastGC data dataset, after data processing, retention time and area under the peak were extracted from chromatograms by manual selection. The baseline of a chromatogram for each mass was calculated according to Sensitive Nonlinear Iterative Peak (Morháč et al. 1997) and was removed for further analysis. The peak position for retention time calculation was obtained by Gaussian method.

From the fastGC PTR-ToF-MS dataset 203 mass peaks were extracted. However, only 25 mass peaks with a chromatographic peak present in at least 50% of the samples were analyzed.

Multivariate statistical analysis was performed using R 3.2.0 internal statistical functions and external packages, namely ggplot2, ChemometricsWithR, VennDiagram, heatmap3, vegan, Peaks.

## Results and discussion

### Efficiency of the method

Additional technical replicates are useful to define the measurement error and biological ones can improve the efficiency of statistical testing (Blainey et al. 2014). The combination of the PTR-ToF-MS and the Autosampler gave the possibility to measure VOCs at a high-throughput level. The overall number of replicates was chosen in order to measure each sample every 4 h. Thus 12 biological replicates for each yeast strain were selected for evaluation of random biological variation of the yeast volatilome. Furthermore, 12 technical replicates of yeast substrate and six empty samples were evaluated for random noise associated with equipment.

Gaus et al. established the general detection efficiency, sensitivity, reproducibility of PTR-ToF-MS measurements (Gaus et al. 2010). The reproducibility of the used PTR-ToF-MS apparatus was controlled by periodic calibrations with a calibration gas standard (Ionicon Analytik GmbH, Innsbruck, Austria). Technical variation within the current dataset was 18.64% (reported as the median spectral RSD for substrate samples). Variation between individuals in one group includes both biological and technical variations. For M28 segregates biological variation ranged from 17.92 to 28.96%, for the laboratory strains it reached 20%.

### Evolution of yeast volatilome during colony development

**Fig. 1:**
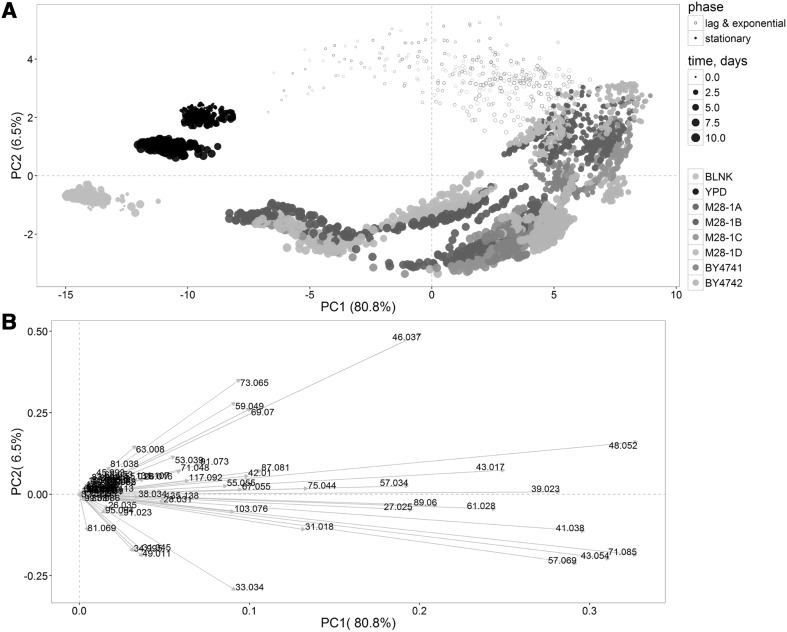
Score plot (**a**) and loading plot (**b**) of principal component analysis of VOC emission evolution for yeasts, medium and blank samples during 11 days of experiment. Data are logarithmically transformed and centered. *Different colors* indicate different yeast strains, medium and blank samples. The size of points grows with time of measurement

In order to evaluate the potential of the technique to characterize and differentiate the metabolic profiles of *S. cerevisiae* during colony development, we selected the well-known and widely used laboratory strains BY4741 and BY4742 together with four meiotic segregants of a natural strain, M28, isolated from Montalcino grapes (Cavalieri et al. 2000). The overall yeast volatilome selected for this study contained 70 mass peaks out of which 50 mass peaks were assigned with a chemical formula and 37 were tentatively identified. The detailed information about the dataset is presented in Supplementary Table I. One of the aims of this study was to explore the evolution of yeast volatilome during colony development. For highlighting of the overall changes in yeast volatilome with time, principal component analysis (PCA) was performed on the dataset of yeast, medium and blank samples VOCs during the 11 days of experiment. Figure 1 shows the evolution of VOCs produced during yeast colony development. The first two principal components explain 87.3% of variability between samples in the dataset, with the first one capturing most of the variance (80.8%). The third principal component only marginally contributes to the explanation of sample variability (6.5%). In order to eliminate the additional variance which could be provoked by medium and blank samples another PCA was performed on the dataset of only yeast VOCs which demonstrated similar results (Supplementary Fig. 4).

The yeast volatilome evolved describing semicircles starting from the top left of the score plot (Fig. 1a) starting near medium samples and finishing at its bottom left side close to blank ones. Empty circles in Fig. 1a correspond to the measurements collected during both the lag and exponential phases which last for one day for the M28 strains and about one and a half day for the laboratory strains (Table 1). Filled circles, on the other hand, represent measurements during the stationary phase. The first principal component shows changes of yeast VOC profiles from left to right during the lag and exponential growth phases due to the rapid colony propagation and in the opposite direction during the stationary phase with a time-dependent decrease in the production of almost all the secondary volatile metabolites. The second principal component, instead, mostly shows the differences between yeast volatilome during lag/logarithmic and stationary phases. Even if the laboratory and natural strains have evident differences in growth (Table 1), the yeast volatilome evolution of the different strains showed a similar trend. This could be ascribed to the fact that the growth effect dominated the difference in the aroma between different strains.

The loading plot presented at Fig. 1b puts in evidence important mass peaks at different stages of yeast colony growth. During the exponential phase (up to one and a half day from the beginning of the experiment) the highest mass peaks in yeast VOC profiles were m/z73.065 (C_4_H_8_OH^+^—tentatively identified (t.i.) as butanal), m/z65.045 (unidentified), m/z59.049 (C_3_H_6_OH^+^—t.i. acetone), m/z69.070 (C_5_H_9_^+^—common fragment), m/z46.037 (C^13^CH_4_OH^+^—t.i. isotope of acetaldehyde). Several mass peaks pointing to the right direction of the loading plot (Fig. 1b) played a major role in explaining the total variance and are also likely related to fermentation processes, i.e. m/z48.052 (C^13^CH_6_OH^+^—t.i. isotope of ethanol). Among other mass peaks pointing in the same direction of ethanol in the PCA loading plot (Fig. 1b) it is possible to find m/z43.017 (C_2_H_3_O^+^—common ester fragment), m/z57.034 (C_3_H_4_OH^+^—common fragment), m/z75.044 (C_3_H_6_O_2_H^+^—t.i. propanoic acid and a fragment of m/z 103.076). The next group of mass peaks contributing to the PCA loading plot includes m/z61.028 (C_2_H_4_O_2_H^+^—tentatively identified as acetic acid and common acetate fragment) and m/z89.060 (C_4_H_8_O_2_H^+^—t.i. ethyl acetate, isobutyric acid, butanoic acid, and/or acetoin). These tentatively identified compounds can exhibit important changes during the growth of yeast colonies.

*Saccharomyces cerevisiae* strains are able to synthesize higher alcohols. Among them we tentatively identified 3-methyl-1-butanol and 2-methyl-1-butanol, isobutyl alcohol and 1-butanol and their common fragments: m/z41.038 (C_3_H_5_^+^—common fragment), m/z43.054(C_3_H_7_^+^—common fragment), m/z71.085 (C_5_H_11_^+^—t.i. dehydrated fragment of alcohols such as 3-methyl-1-butanol and 2-methyl-1-butanol), m/z57.069 (C_4_H_9_^+^—t.i. a dehydrated fragment of butanol isomers).

It is clearly seen from Fig. 1b that four mass peaks such as m/z33.034 (CH_4_OH^+^—t.i. methanol), m/z49.011 (CH_4_SH^+^—t.i. methanethiol), m/z31.045 (unidentified), m/z34.995 (H_2_SH^+^—t.i. hydrogen sulfide) started playing an important role close to the end of the experiment.

### *S. cerevisiae* characterization

#### Curve investigation

Real time monitoring of VOCs was performed to investigate deeply the differences in yeast volatilome of selected strains and find possible explanations from genetic and phenotypical point of view. The laboratory strains BY4741 and BY4742 differ genetically only in the deletion of LYS2 and MET15 and by opposite mating type. Four meiotic segregants of the same natural strain, M28, were selected to investigate how much volatilome variation could be embedded in heterozygosity in any given wild strain. Albeit deriving from the same parental strain, the four meiotic segregants differ in two macroscopic phenotypes: (i) M28-1A and M28-1D form smooth colonies, while M28-1B and M28-1C form filigreed colonies; (ii) segregants M28-1A and M28-1C are sensitive to 5′,5′,5′-trifluoroleucine (TFL), while the segregants M28-1B and M28-1D are resistant to TFL due to a mutation in the Ssy1 gene (Brown et al. 2008). BY4741 and BY4742 are sensitive to TFL as well.

**Fig. 2:**
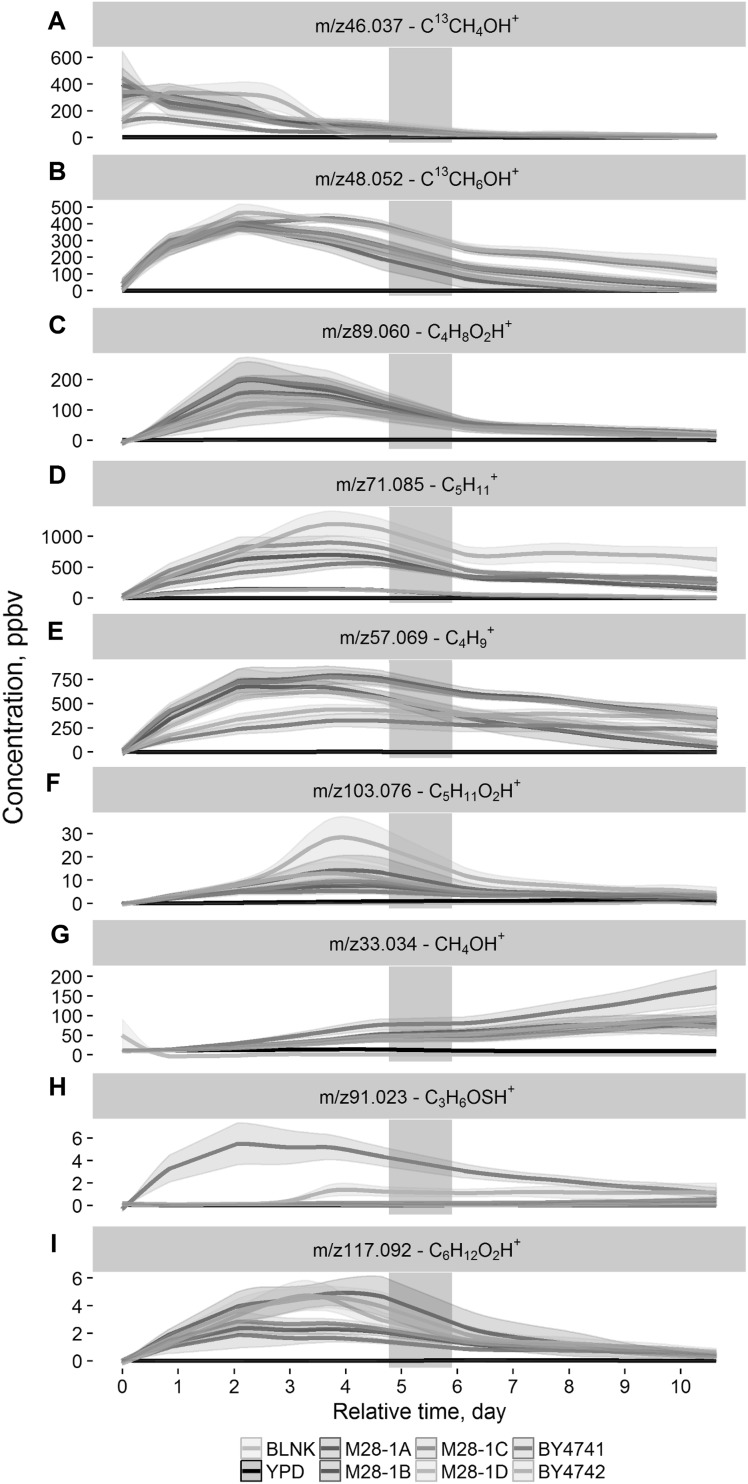
Curves of selected mass peaks tentatively identified as an isotope of acetaldehyde (**a**), an isotope of ethanol (**b**), ethyl acetate, isobutyric acid, butanoic acid, and acetoin (**c**), a dehydrated fragment of alcohols such as 3-methyl-1-butanol and 2-methyl-1-butanol (**d**), a dehydrated fragment of butanol isomers (**e**), isovaleric acid, ethyl propanoate, 2-methylbutanoic acid (**f**), methanol (**g**), S-methyl thioacetate (**h**), and ethyl butyrate, ethyl isobutyrate, isobutyl acetate **(**i**)**. Curves of each sample represent mean value and standard error of each yeast strain, medium and blank for each time point upon smoothing. *Grey rectangles* show the period when samples were measured with fastGC PTR-ToF-MS

Figure 1 captures the general picture of yeast colonies’ VOC profiles evolution during the 11 days showing the drastic differences with growth. To investigate the effect of growth deeply, nine mass peaks were selected for a detailed visualization of the time course. The relative plots are shown in Fig. 2. To improve clarity, a breakdown of the nine time courses is in high resolution is included in supplementary materials. The first seven mass peaks (Fig. 2a–g) were selected from PCA as they are the major players in the yeast volatilome evolution. The other two are interesting because of their particular behavior which will be described in detail further.

Figure 2a (Supplementary Fig. 5) shows the evolution of acetaldehyde (m/z46.037) as an example of the compounds showing the decreasing trend such as m/z73.065 (C_4_H_8_OH^+^—tentatively identified (t.i.) as butanal), m/z65.045 (unidentified), m/z59.049 (C_3_H_6_OH^+^—t.i. acetone), m/z69.070 (C_5_H_9_^+^—common fragment). In particular, the profile of acetaldehyde highlights the differences in the growth profile observed between laboratory and natural strains. Indeed, while all the four meiotic segregants of the M28 strains displayed an initial high level of acetaldehyde, followed by a gradual decrease, both BY strains (especially BY4742) showed an initial lower level of acetaldehyde followed by a peak of emission during the first days of growth. In the first 3 days the acetaldehyde profile of BY4742 reached a level similar to the one produced by the M28’s meiotic segregants, while in BY4741 the acetaldehyde profile underwent a temporary increase, whose maximum was significantly lower than the maximum produced by the other strains. Acetaldehyde is one of the first compounds produced during glucose fermentation, and tends to accumulate within the cells if not further metabolized (Stanley and Pamment 1993). The observed trend is thus an indication of the strain growth. The variance in the total amount of acetaldehyde measured from strains having different genetic background was previously observed and is exacerbated when growing strains at 30 °C (Romano et al. 1994). Nevertheless, it was surprising to observe a noticeable distinction among closely genetically related strains with the opposite mating type (the BYs strains).

The curve of ethanol (m/z48.052) evolution is presented in Fig. 2b (Supplementary Fig. 6). For the four M28 segregants ethanol production reached its maximum during the 2nd day. The subsequent decrease of this compound was faster for M28-1B and M28-1D than for M28-1A and M28-1C. Both BY4741 and BY4742 were able to produce ethanol for longer time than the M28 strains. The particular growth characteristic of each yeast strain can probably explain these differences.

Figure 2c (Supplementary Fig. 7) demonstrates the changes of m/z89.060 with time. This mass peak was tentatively identified as ethyl acetate, isobutyric acid, butanoic acid, and/or acetoin. According to Saerens et al. 2008 ethyl acetate is produced inside yeast cells during fermentation and can diffuse through cell membrane into medium and headspace rapidly and completely. In addition, several of these compounds have been shown to hold a fundamental role in the strain’s ability to attract insects, a process through which *S. cerevisiae* likely relies to be dispersed among different environments, thus being relevant for the ecology of natural strains (Christiaens et al. 2014).

For the production of higher alcohols Fig. 2d (Supplementary Fig. 8) shows that the TFL-resistant M28-1B and M28-1D strains produced significantly lower concentrations of 3-methyl-1-butanol and 2-methyl-1-butanol compared to the other M28 strains and the two laboratory strains. This observation can be ascribed to a frameshift mutation in the Ssy1 gene resulting in defective amino acids transport across the plasma membrane with the consequent up regulation of amino acids biosynthetic pathways as a compensatory cellular mechanism (Brown et al. 2008). Actually, the block of amino acids import in the TFL-resistant strains could explain the reduction of 3-methyl-1-butanol levels in M281B/D since this metabolite is normally produced via Ehrlich pathway from leucine and valine catabolism (Park et al. 2014), in agreement with the knowledge that production of amino acids within the cell in Ssy1 mutants reaches lower amino acid levels than those normally supplemented in the growth medium (Klasson et al. 1999). Figure 2e (Supplementary Fig. 9) illustrates the evolution of butanol with time. During the first 3 days the level of butanol was slightly lower in the segregants resistant to trifluoroleucine than in sensitive ones. However, with time this difference increased. A new pathway for the biosynthesis of n-butanol requiring glycine as substrate has been recently proposed, and this could be the explanation of the low amounts of n-butanol in TFL-resistant strains where amino acid uptake is strongly impaired (Branduardi et al. 2013). The curve of butanol grew more rapidly than the one of 3-methyl-1-butanol and 2-methyl-1-butanol. Supplementary Fig. 10 represents the correlation between the emissions of these alcohols. The curves placed under the diagonal line emitted faster and higher concentration of butanol that 3-methyl-1-butanol and 2-methyl-1-butanol.

The mass peak m/z103.076 (C_5_H_10_O_2_H^+^—t.i. isovaleric acid, ethyl propanoate, 2-methylbutanoic acid) reached its maximum at the fourth day as it is shown in Fig. 2f (Supplementary Fig. 11). To note, this compound was produced by BY4742 strain almost at a double rate than by the other strains. Isovaleric acid is produced from leucine through the Ehrlich pathway (Park et al. 2014), while ethyl propanoate and 2-methylbutanoic acid form by the reaction of ethanol with a fatty acid (Saerens et al. 2010). Despite these compounds are known to be produced during yeast fermentations, the fact that we unexpectedly observed different levels in their production dynamics suggests that either the feedback inhibition sensitivity or the transport of amino acids present in the media differs significantly in different strains.

Figure 2g (Supplementary Fig. 12) shows that methanol concentration grows with colony ageing. BY4741 produces more methanol than others, probably because of the MET15 deletion which is typical for this strain (Thomas and Surdin-kerjan 1997). Moreover, three mass peaks were tentatively identified as sulfur compounds such as m/z34.995 (H_2_SH^+^—t.i. hydrogen sulfide), m/z49.011 (CH_4_SH^+^—t.i. methanethiol), m/z91.023 (C_3_H_6_OSH^+^—t.i. S-methyl thioacetate). The distinctive feature of these compounds was their higher emission by BY strains than by M28 ones. As it is shown in Fig. 2h (Supplementary Fig. 13) the production of these compounds started earlier for BY4741 strain than for BY4742 reaching highest values between the second and the fourth day with a subsequent decrease. The active emission of these compounds by BY4742 started at the 4th day and was mostly constant during the following days. MET15 codes for the O-acetyl homoserine-O-acetyl serine sulfhydrylase which catalyses the reaction between acetylated serine or homoserine with thiol to produce homocysteine or with methanethiol to produce l-methionine (Thomas and Surdin-kerjan 1997). It is very likely that deletion of this gene in the BY4741 strain is the main factor responsible of hydrogen sulfide and methanethiol accumulation, since they are not used as substrate.

#### Investigation of a single time point

**Fig. 3:**
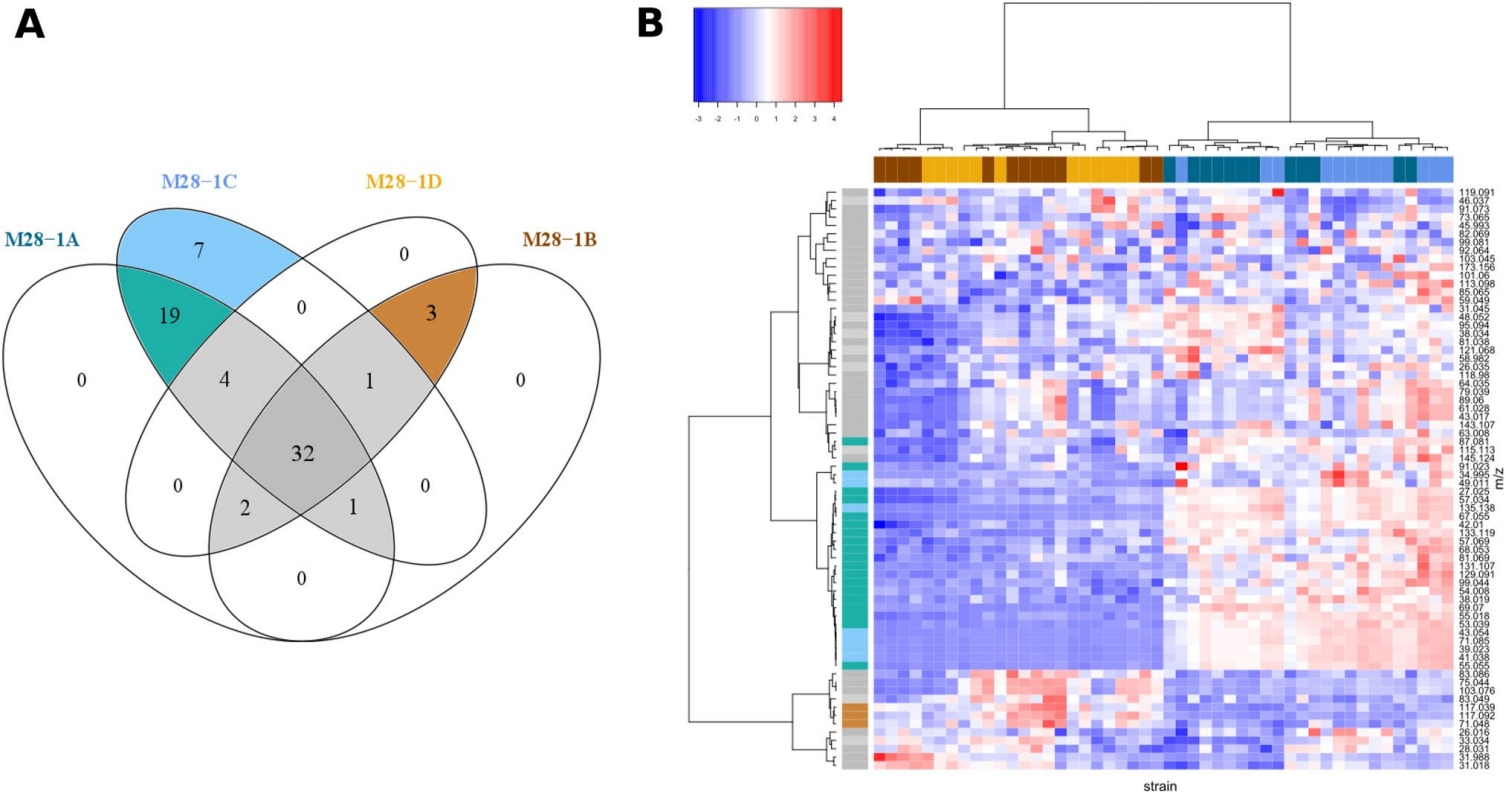
Venn diagram (**a**) of the 69 mass peaks which are grouped according to their concentration range in VOC profiles of the four meiotic segregants of M28 natural strain (p < 0.001, Tukey’s test) and heat map (**b**) of unsupervised hierarchical clustering of the centered and scaled data in both mass peaks (*rows*) and replicates of the M28 strain (*columns*). *Colors of the vertical side bar* correspond to mass peak types presented at Venn diagram (**a**). *Colors of the horizontal side bar* annotate M28 strain

Another objective of this work was to investigate the differences between each yeast strain at a specific time point. For this reason one whole cycle of measurements of all 90 samples after 108 h (4 days and a half) from the beginning of the experiment was selected. At this point, the rapid growth of the colonies is finished, but they are still not declining. The chosen interval corresponds to one cycle before the measurement with fastGC. In order to focus on the features of the four meiotic segregants of M28 natural strain and the two laboratory strains, further analysis was performed separately for these two groups. Venn diagram presented at Fig. 3a shows that 40 out of 69 mass peaks do not differ significantly for at least three meiotic segregants out of four. It also confirms that M28-1A and M28-1C in general produce more VOCs. Seven mass peaks such as m/z34.995 (H_2_SH^+^—t.i. hydrogen sulfide), m/z39.023 (C_3_H_3_^+^—common fragment), m/z41.038 (C_3_H_5_^+^—common fragment), m/z43.054 (C_3_H_7_^+^—common fragment), m/z49.011 (CH_4_SH^+^—t.i. methanethiol), m/z71.085 (C_5_H_11_^+^—t.i. dehydrated fragment of alcohols such as 3-methyl-1-butanol and 2-methyl-1-butanol), m/z135.138 (unidentified) were significantly higher in M28-1C only. Moreover, it was found that M28-1B and M28-1D could be characterized by significantly higher emission of m/z71.048 (C_4_H_6_OH+—t.i. butenal), m/z117.039 (unidentified), m/z117.092 (C_6_H_12_O_2_H^+^—t.i. ethyl butyrate, ethyl isobutyrate, isobutyl acetate) as confirmed by Fig. 2i (Supplementary Fig. 14). It is important to mention that these two strains are characterized by their resistance to TFL, characteristics that has been shown to impact also on the production of volatile compounds (Casalone et al. 1997). In previous studies an increased production of isoamyl alcohol only was found in resistant strains. Nevertheless, it has to be considered that this is the first untargeted assessment of their complete volatilome, thus these results could indicate effects on different metabolic pathways.

**Fig. 4:**
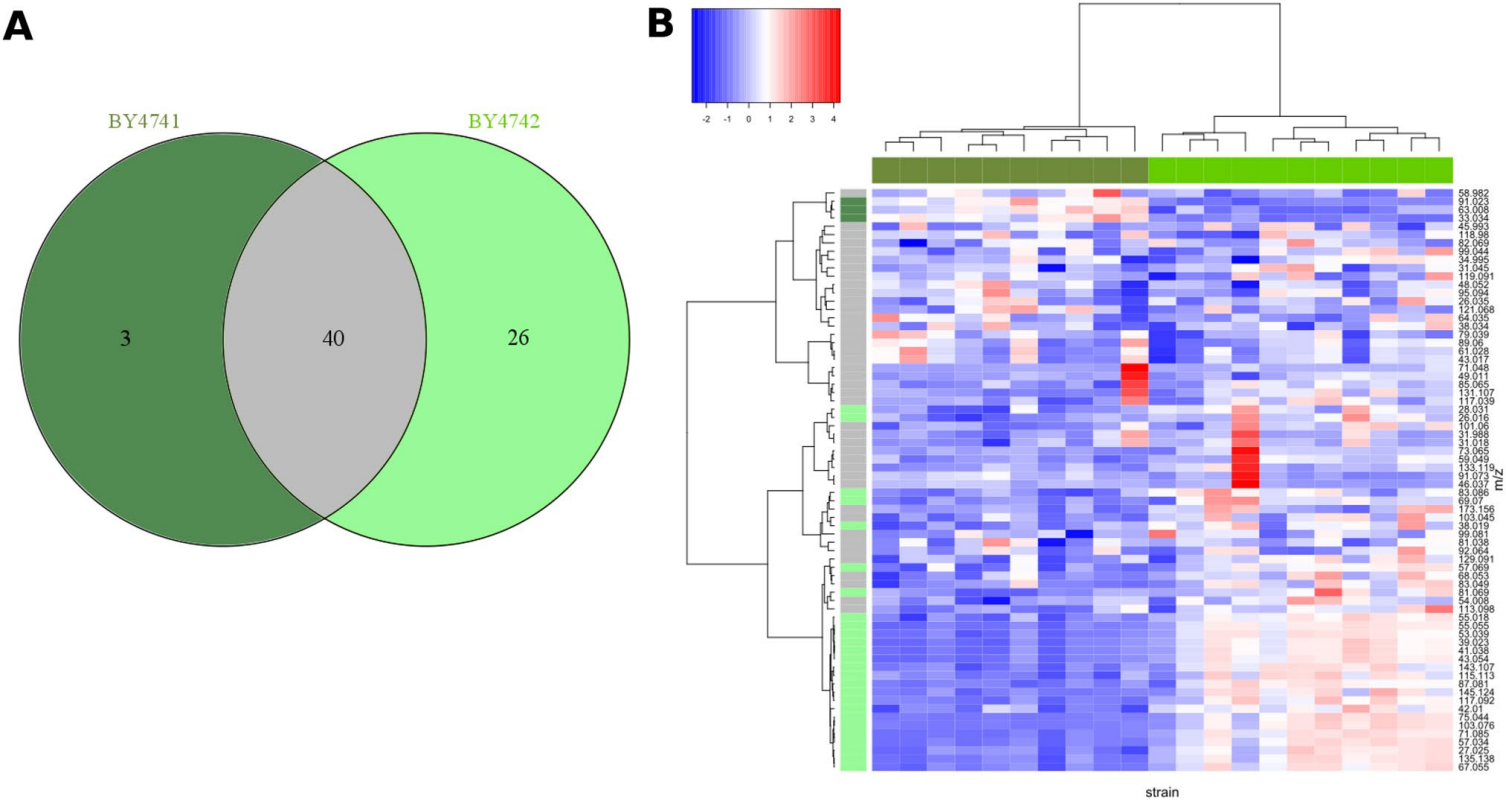
Venn diagram (**a**) of the 69 mass peaks which are grouped according to their concentration range in VOC profiles of the two laboratory strains BY4741 and BY4742 (p < 0.001, Tukey’s test) and heat map (**b**) of unsupervised hierarchical clustering of the centered and scaled data in both mass peaks (*rows*) and replicates of the strains (*columns*). *Colors of the vertical side bar* correspond to mass peak types presented at Venn diagram (**a**). *Colors of the horizontal side bar* annotate the strains

According to the Venn diagram (Fig. 4a) and the heat map (Fig. 4b), BY4742 emits in general much more VOCs in comparison to BY4741. However BY4741 can be distinguished from BY4742 by higher concentration of m/z33.034 (CH_4_OH^+^—t.i. methanol, Fig. 2g), m/z63.008 (unidentified), and m/z91.023 (C_3_H_6_OSH^+^—t.i. S-methyl thioacetate, Fig. 2h). The levels of other two sulfur compounds such as m/z34.995 (H_2_SH^+^—t.i hydrogen sulfide) and m/z49.011 (CH_4_SH^+^—t.i. methanethiol) were significantly higher for BY4741 during the first 3 days of yeast growth as for m/z91.023. However, from the third till the 6th day BY4742 emitted those two compounds in significantly higher concentrations than BY4741. These results reflected what previously observed in general in the latest PTR-ToF-MS data, with the BY4742 strain accumulating high amounts of metanethiol as a consequence of the MET17 gene deletion.

### *Saccharomyces cerevisiae* distinction by fastGC PTR-ToF-MS

**Fig. 5:**
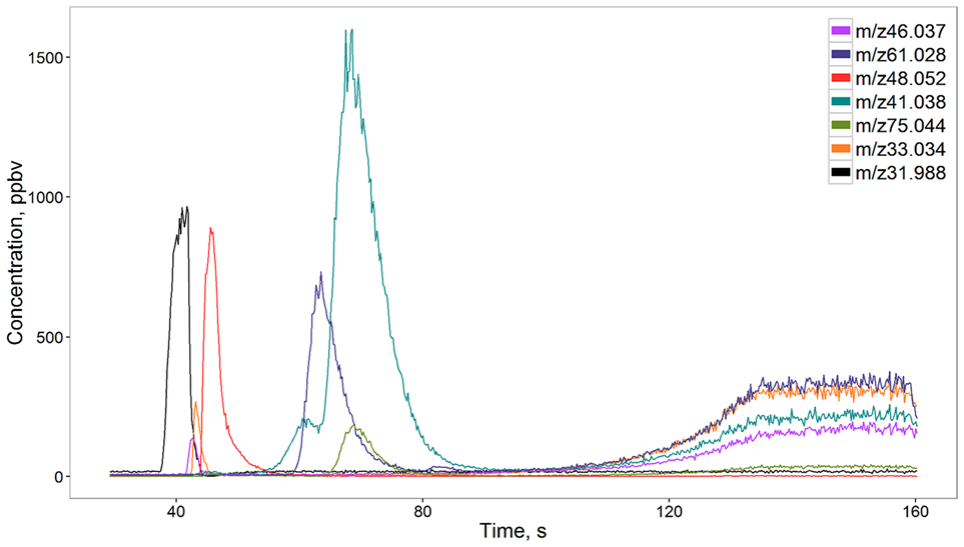
Example of a yeast chromatogram obtained by fastGC PTR-ToF-MS

In processed data obtained by coupling fastGC with PTR-ToF-MS we found less mass peaks than just with PTR-ToF-MS. Figure 5 shows the nine peaks present in a yeast sample chromatogram made by fastGC PTR-ToF-MS. Column bleed was observed at temperatures higher than 200 °C. Each peak in the chromatogram represents a compound emitted by yeasts, injected in a fastGC column and eluted from it. The first peak belongs to oxygen (O_2_^+^—m/z31.988), which does not interact with the analytic column and is used as a reference peak. Acetaldehyde (C^13^CH_4_OH^+^—m/z46.037) and methanol (CH_4_OH^+^—m/z33.034) elute before ethanol. Due to fastGC features it was possible to measure high concentrations of ethanol and to investigate its fragmentation and cluster pattern without any dilution of the sample headspace. Mass peaks such as m/z48.052 (C^13^CH_6_OH^+^—t.i. isotope of ethanol), m/z29.039, m/z30.044 (C_2_H_5_^+^—t.i. fragment of ethanol and its isotope), m/z65.059 (C_2_H_6_O·H_3_O^+^—t.i. ethanol cluster), m/z75.081 (C_2_H_5_OH·C_2_H_5_^+^—t.i. ethanol cluster), m/z93.0904 (C_2_H_6_O·C_2_H_6_OH^+^—t.i. ethanol cluster) were observed during ethanol elution. FastGC data confirmed the results obtained with PTR-ToF-MS alone. Due to the possibility of fastGC to analyze samples with high concentration of ethanol it was verified that at the fifth day of experiment BY4741 and BY4742 emit more ethanol than the M28 segregants.

**Fig. 6:**
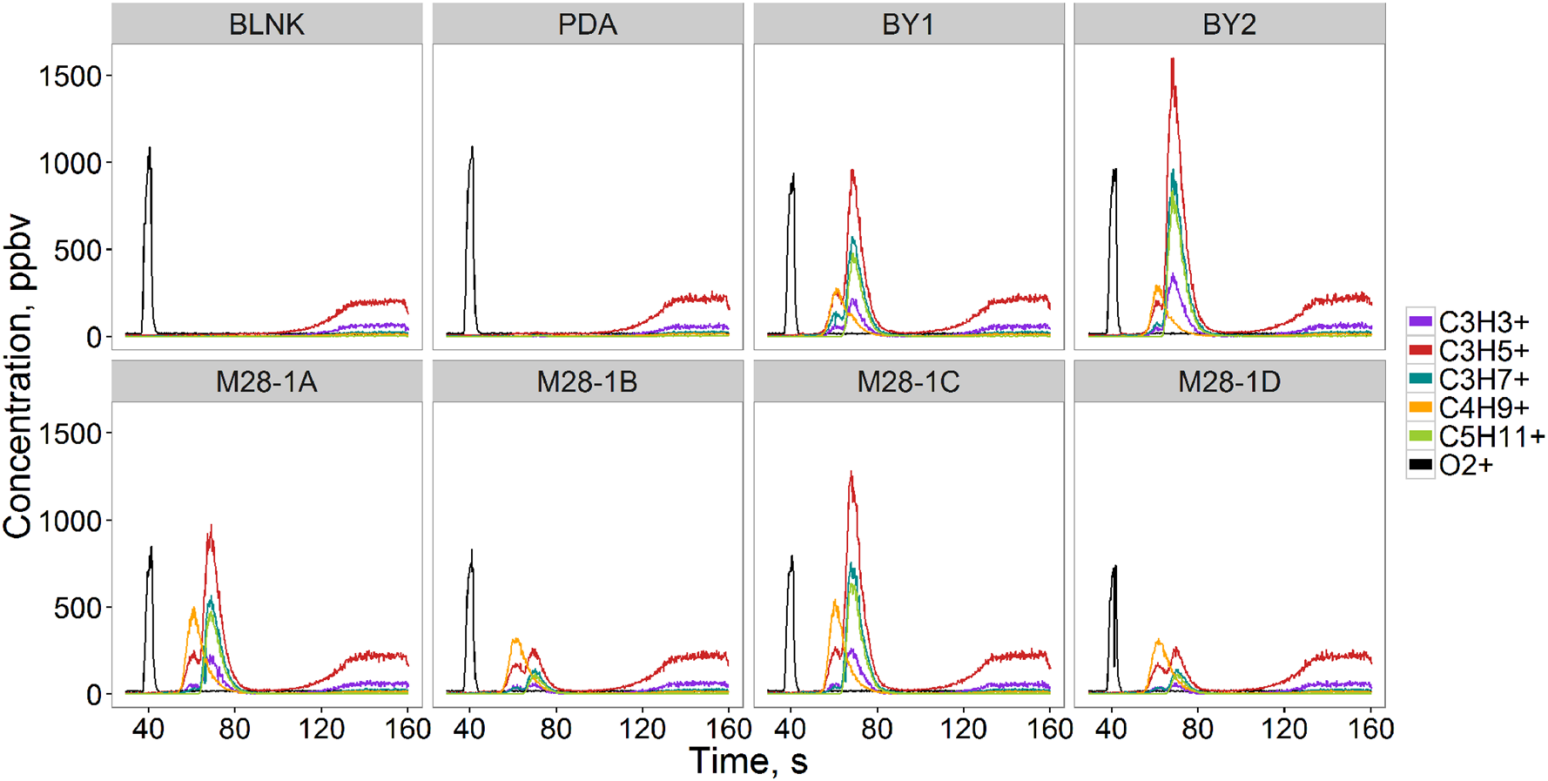
Selected region of the chromatogram of butanol and amyl alcohols for each yeast strain, medium and blank samples. Each *color* represents a different mass peak

Common fragments such as m/z39.023 (C_3_H_3_^+^), m/z41.038 (C_3_H_5_^+^), m/z43.054 (C_3_H_7_^+^) form a double peak at the fastGC chromatogram (Fig. 6), representing two different compounds which are not distinguishable by only PTR-ToF-MS measurements. The first local maximum of this peak is also shared with m/z57.069, which most probably belongs to isobutanol according to the retention time. Some trace peak of most 1-butanol was found to the right from the first apex. The second one is instead shared with m/z71.085 (C_5_H_11_^+^—fragment of alcohols such as 3-methyl-1-butanol and 2-methyl-1-butanol). FastGC PTR-ToF-MS also allowed to distinguish between two isomers such as acetic acid and the fragment of ethyl acetate (C_2_H_4_O_2_H^+^—m/z61.028) which has shorter retention time.

## Conclusions

In this work yeast volatilome changes during colony growth were monitored in a rapid, real-time and nondestructive way during the whole fermentation process. PTR-ToF-MS coupled to a multipurpose GC autosampler guaranteed the high throughput, reproducibility and automatization of the experiment. It was possible to trace the evolution of various VOCs and to characterize two laboratory strains and four meiotic segregants of a natural strain isolated from wine must fermentation according to their VOC profiles. Remarkably, significantly different concentration levels of 3-methyl-1-butanol and 2-methyl-1-butanol were found between TFL-sensitive and TFL-resistant meiotic segregants of the M28 yeast strain. Peculiar differences in methanol and several sulfur compounds were observed in the laboratory strains. Moreover, a fastGC add-on coupled to PTR-ToF-MS enriched the current investigation with the data of an additional gas chromatographic dimension without considerable increase in measurement time. This novel setup was useful in the separation of several isomeric compounds which were found in yeast VOC profiles and were inseparable for PTR-ToF-MS without a fast GC add-on. Despite the high genetic similarity within the two groups of tested strains (BY and M28 series), it was possible to unveil the metabolic differences among these strains and highlight previously unobserved strain-related patterns. The increased levels of volatiles by products of amino acid metabolism could reflect the increased ability of the By series strains to exploit externally supplemented amino acids, potentially as an adaptation to the conditions present in the laboratory. This trait potentially could be resulting from the selection procedure that led to establish the father of By strains, S288c as the model for *S. cerevisiae* (Mortimer and Johnston 1998).

The possibility to screen strains in solid culture and the possibility to automate the process could enable in the future, rapid processing of large yeast collections in a timely and low cost manner. Quality control and stability assessment of strains in collections is a problem that has accompanied the development of yeast microbiology. The current method could be proposed as a fast method of choice to phenotype industrial strains. Thus, the ability demonstrated in discriminating genetically very similar strains, suggests this as also as a method of choice for strain phenotyping, identification, and quality control.

Furthermore, this technique holds thus the potential to greatly help in the complete dissection of new metabolic pathways and regulations at a wider level and in the disclosure of the complete phenotypic variability among natural and laboratory *S. cerevisiae* strains. In addition to the impact on the understanding of yeast metabolic pathways, the findings are also relevant for the potential implications in the exploitation of the microbial diversity in wine, beer, and bread productions and, more generally, for the investigation and monitoring of bioprocessing.

## Electronic supplementary material

Below is the link to the electronic supplementary material.

Supplementary Figure 1—Schematic representation of the experimental setup (Headspace measurement: Black solid and square dot arrows show the order of their usage. Measurements with fastGC follow the black and grey square dot arrows) (JPEG 81 KB)

Supplementary Figure 2—Schematic drawing of PTR-ToF-MS inlet system with a fastGC add-on. The updated version of fastGC setup with a separated line for measurements while fastGC is disable (JPEG 218 KB)

Supplementary Figure 3—FastGC thermal ramp (PNG 35 KB)

Supplementary Figure 4—Score plot (A) and loading plot (B) of principal component analysis of VOC emission evolution for yeasts and medium samples during 11 days of experiment. Data are logarithmically transformed and centered. Different colors indicate different yeast strains (PNG 567 KB)

Supplementary Figure 5—Curves of m/z46.037 (C^13^CH_4_OH^+^ - tentatively identified as an isotope of acetaldehyde) of yeast strains, medium and blank samples. Curves of each sample represent mean value and standard error of each sample type for each time point upon smoothing. Grey rectangle shows the period when samples were measured with fastGC PTR-ToF-MS. This figure corresponds to Figure 2A (PNG 177 KB)

Supplementary material 6—Curves of m/z48.052 (C^13^CH_6_OH^+^ - tentatively identified as an isotope of ethanol) of yeast strains, medium and blank samples. Curves of each sample represent mean value and standard error of each sample type for each time point upon smoothing. Grey rectangle shows the period when samples were measured with fastGC PTR-ToF-MS. This figure corresponds to Figure 2B (PNG 273 KB)

Supplementary Figure 7—Curves of m/z89.060 (C_4_H_8_O_2_H^+^ - tentatively identified as ethyl acetate, isobutyric acid, butanoic acid, and acetoin) of yeast strains, medium and blank samples. Curves of each sample represent mean value and standard error of each sample type for each time point upon smoothing. Grey rectangle shows the period when samples were measured with fastGC PTR-ToF-MS. This figure corresponds to Figure 2C (PNG 229 KB)

Supplementary Figure 8—Curves of m/z71.085 (C_5_H_11_^+^ - tentatively identified as a fragment of alcohols such as 3-methyl-1-butanol and 2-methyl-1-butanol) of yeast strains, medium and blank samples. Curves of each sample represent mean value and standard error of each sample type for each time point upon smoothing. Grey rectangle shows the period when samples were measured with fastGC PTR-ToF-MS. This figure corresponds to Figure 2D (PNG 181 KB)

Supplementary Figure 9—Curves of m/z57.069 (C_4_H_9_^+^ - tentatively identified as a fragment of butanol isomers) of yeast strains, medium and blank samples. Curves of each sample represent mean value and standard error of each sample type for each time point upon smoothing. Grey rectangle shows the period when samples were measured with fastGC PTR-ToF-MS. This figure corresponds to Figure 2E (PNG 234 KB)

Supplementary Figure 10—Scatter plot of m/z57.069 (C_4_H_9_^+^ - tentatively identified as a dehydrated fragment of butanol isomers) and m/z71.085 (C_5_H_11_^+^ - tentatively identified as a dehydrated fragment of alcohols such as 3-methyl-1-butanol and 2-methyl-1-butanol) of yeast strains, medium and blank samples. Curves of each sample represent mean value of each sample type for each time point upon smoothing. The gap in the lines corresponds to the measurement by fastGC PTR-ToF-MS. The inclination of the curves compares the emission of these two mass peaks (PNG 127 KB)

Supplementary Figure 11—Curves of m/z103.076 (C_5_H_10_O_2_H^+^ - tentatively identified as isovaleric acid, ethyl propanoate, 2-methylbutanoic acid) of yeast strains, medium and blank samples. Curves of each sample represent mean value and standard error of each sample type for each time point upon smoothing. Grey rectangle shows the period when samples were measured with fastGC PTR-ToF-MS. This figure corresponds to Figure 2F (PNG 172 KB)

Supplementary Figure 12—Curves of m/z33.034 (CH_4_OH^+^ - tentatively identified as methanol) of yeast strains, medium and blank samples. Curves of each sample represent mean value and standard error of each sample type for each time point upon smoothing. Grey rectangle shows the period when samples were measured with fastGC PTR-ToF-MS. This figure corresponds to Figure 2G (PNG 150 KB)

Supplementary Figure 13—Curves of m/z91.023 (C_3_H_6_OSH^+^ - tentatively identified as S-methyl thioacetate) of yeast strains, medium and blank samples. Curves of each sample represent mean value and standard error of each sample type for each time point upon smoothing. Grey rectangle shows the period when samples were measured with fastGC PTR-ToF-MS. This figure corresponds to Figure 2H (PNG 126 KB)

Supplementary Figure 14—Curves of m/z117.092 (C_6_H_12_O_2_H^+^ - tentatively identified as ethyl butyrate, ethyl isobutyrate, isobutyl acetate) of yeast strains, medium and blank samples. Curves of each sample represent mean value and standard error of each sample type for each time point upon smoothing. Grey rectangle shows the period when samples were measured with fastGC PTR-ToF-MS. This figure corresponds to Figure 2I (PNG 236 KB)

Supplementary Table 1—VOC significantly different from blank samples and also significantly different either between M28 segregants or between BY4741 and BY4742. Symbol ‘*’ represts compounds found during measurements of one sample from each yeast strain with GC-MS (DOCX 74 KB)
