# Supplementary figures and images for: Non-invasive real time monitoring of yeast volatilome by PTR-ToF-MS
Source: Metabolomics. 2017 Aug 31;13(10):118. doi: 10.1007/s11306-017-1259-y (PMC5579147; doi:10.1007/s11306-017-1259-y)

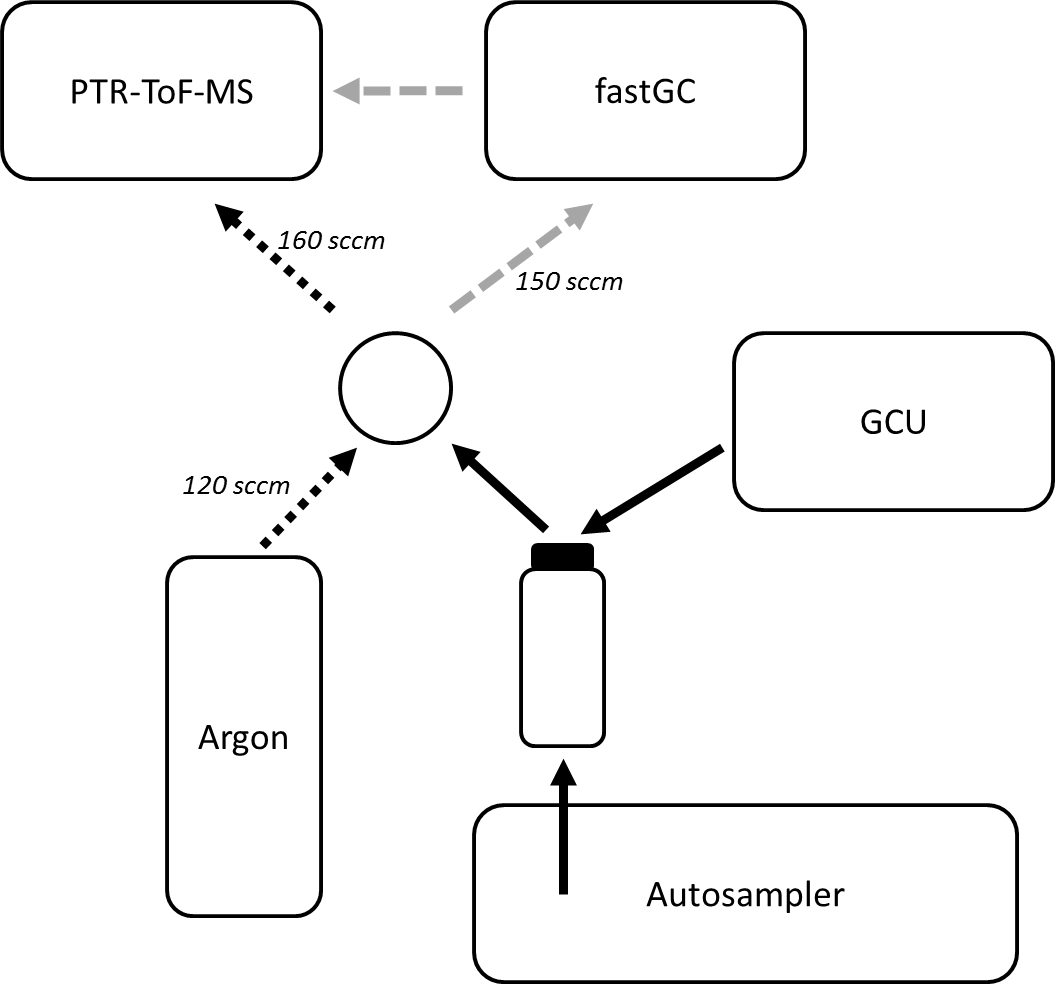

Supplement: Supplementary file 1 — Supplementary Figure 1—Schematic representation of the experimental setup (Headspace measurement: Black solid and square dot arrows show the order of their usage. Measurements with fastGC follow the black and grey square dot arrows) (JPEG 81 KB) [file 11306_2017_1259_MOESM1_ESM.jpeg]

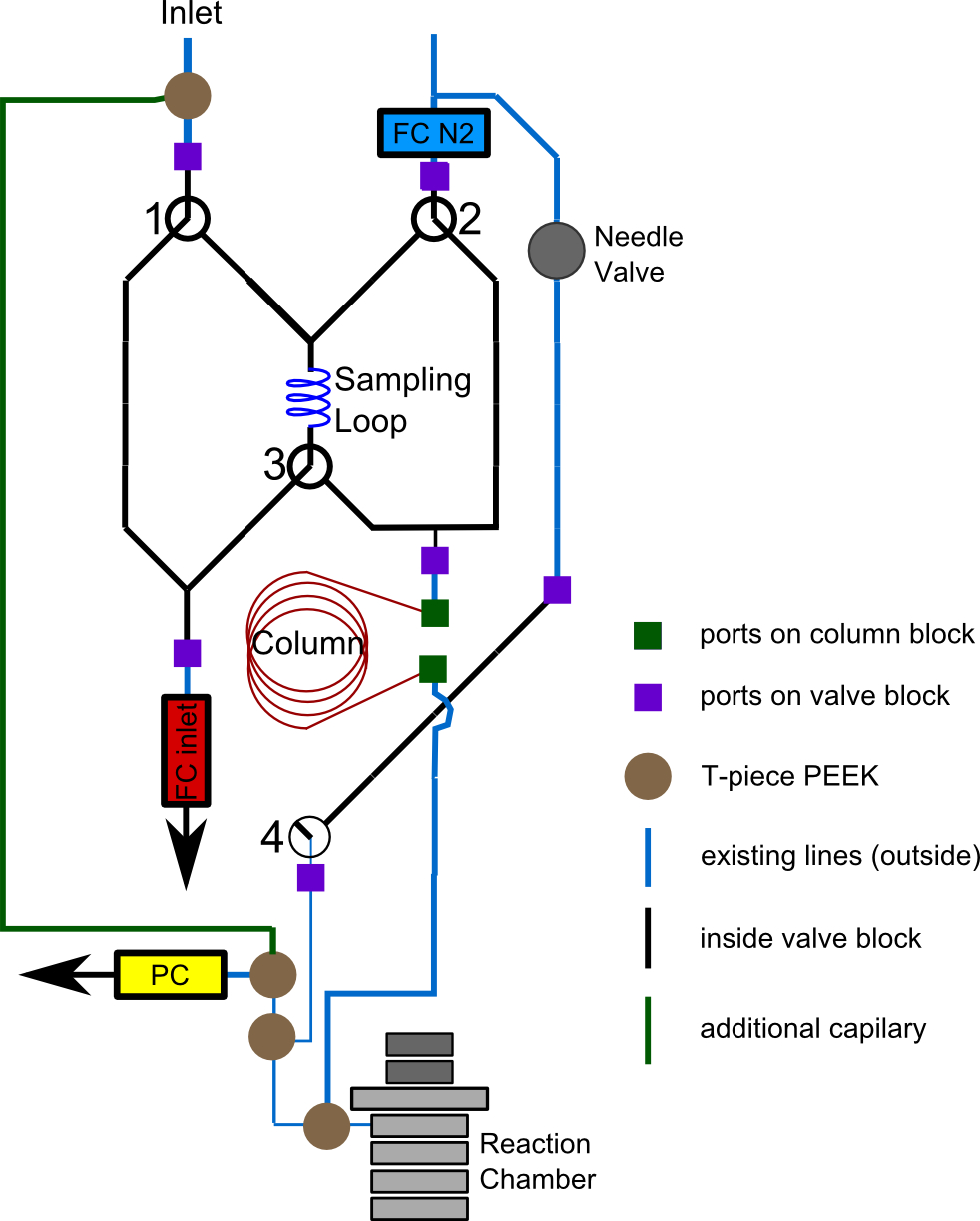

Supplement: Supplementary file 2 — Supplementary Figure 2—Schematic drawing of PTR-ToF-MS inlet system with a fastGC add-on. The updated version of fastGC setup with a separated line for measurements while fastGC is disable (JPEG 218 KB) [file 11306_2017_1259_MOESM2_ESM.jpeg]

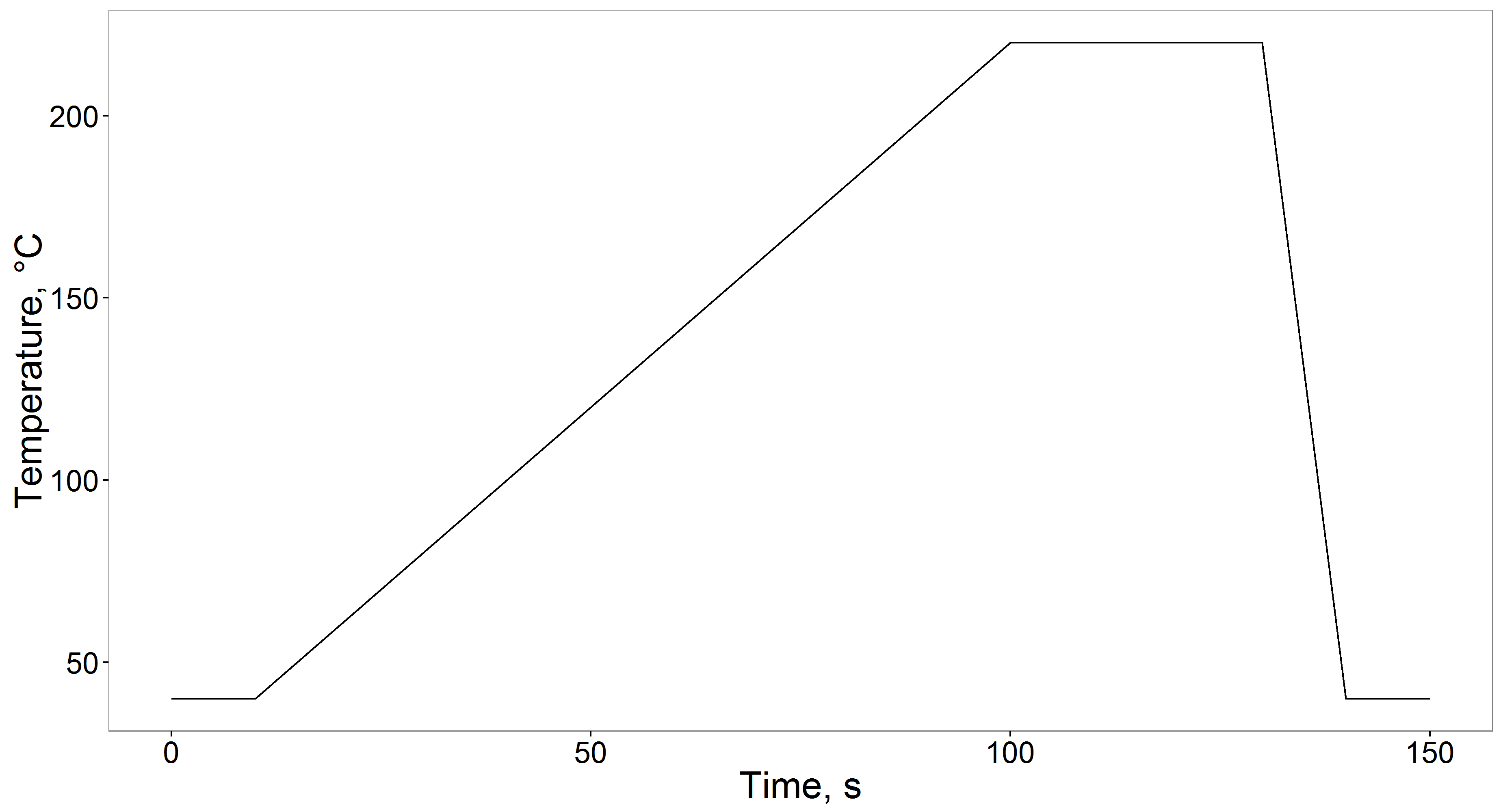

Supplement: Supplementary file 3 — Supplementary Figure 3—FastGC thermal ramp (PNG 35 KB) [file 11306_2017_1259_MOESM3_ESM.png]

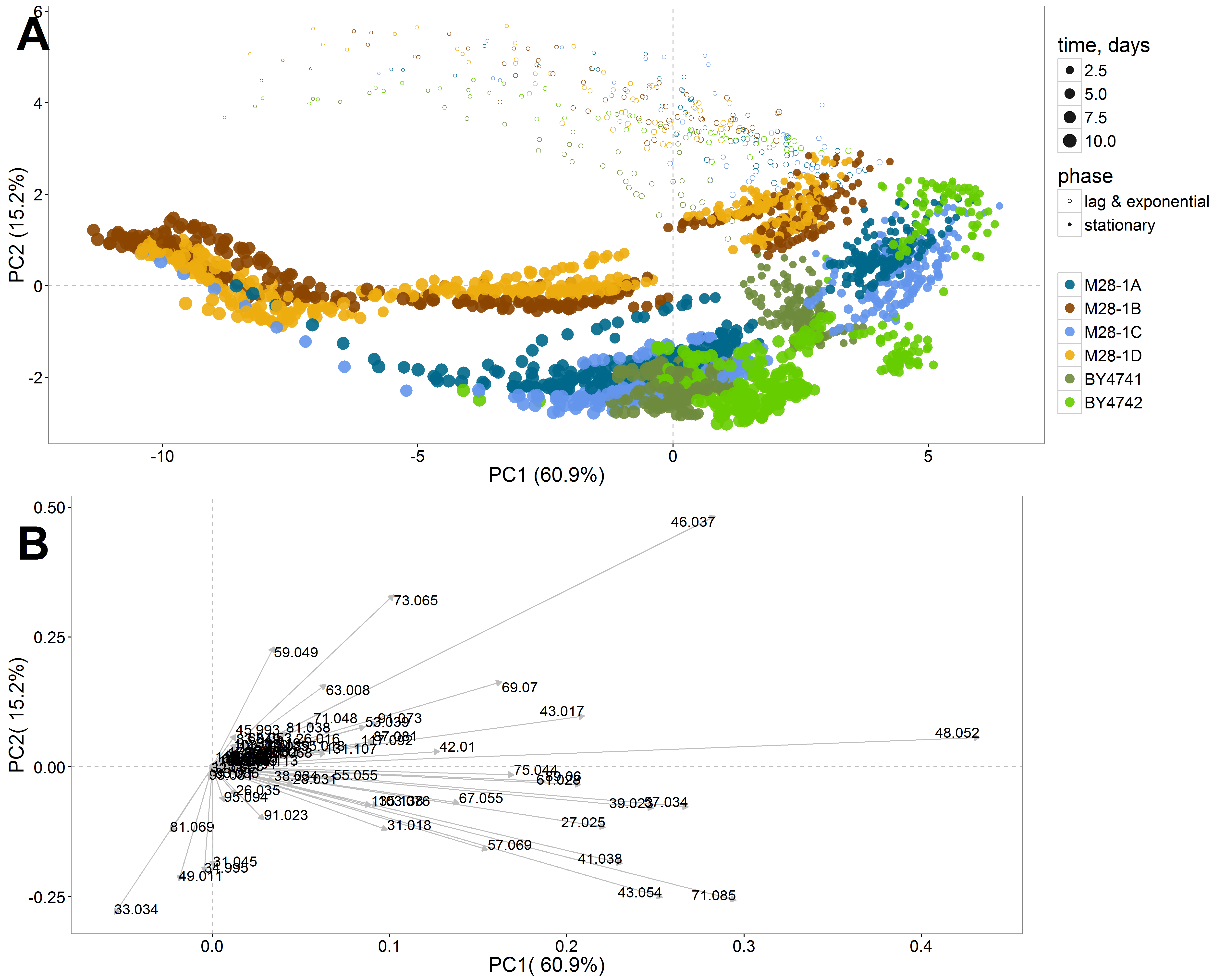

Supplement: Supplementary file 4 — Supplementary Figure 4—Score plot (A) and loading plot (B) of principal component analysis of VOC emission evolution for yeasts and medium samples during 11 days of experiment. Data are logarithmically transformed and centered. Different colors indicate different yeast strains (PNG 567 KB) [file 11306_2017_1259_MOESM4_ESM.png]

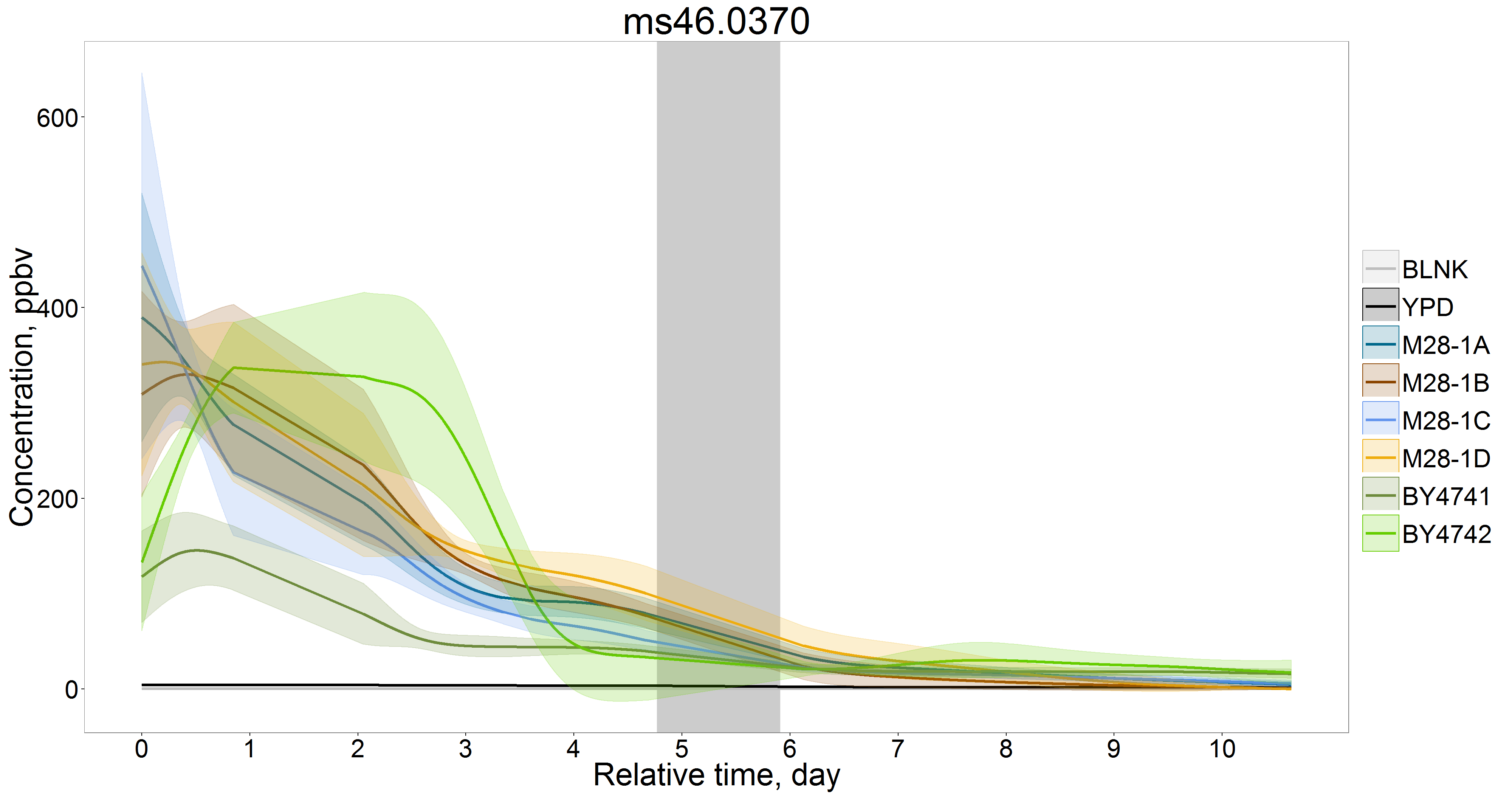

Supplement: Supplementary file 5 — Supplementary Figure 5—Curves of m/z46.037 (C13CH4OH+ - tentatively identified as an isotope of acetaldehyde) of yeast strains, medium and blank samples. Curves of each sample represent mean value and standard error of each sample type for each time point upon smoothing. Grey rectangle shows the period when samples were measured with fastGC PTR-ToF-MS. This figure corresponds to Figure 2A (PNG 177 KB) [file 11306_2017_1259_MOESM5_ESM.png]

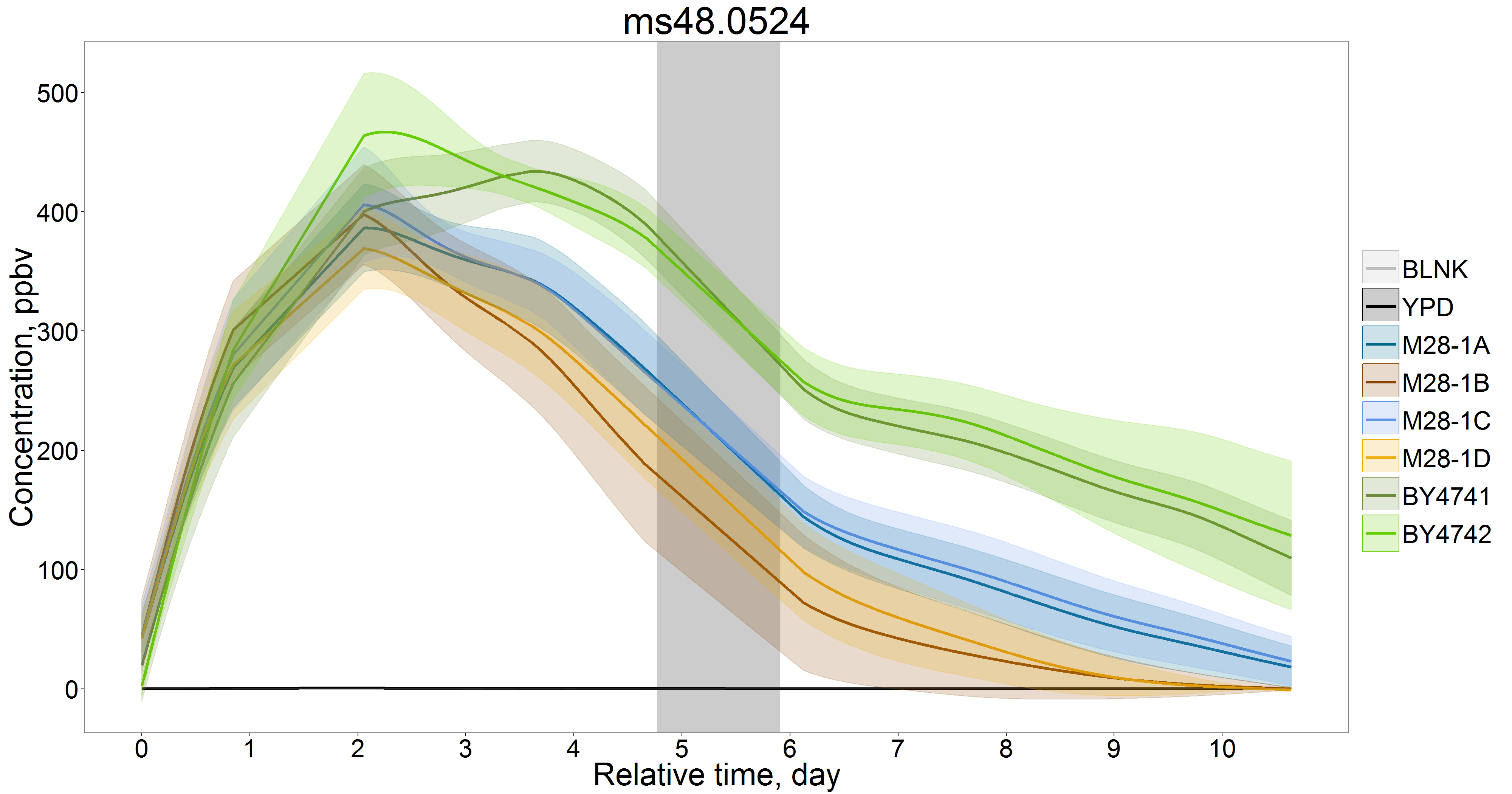

Supplement: Supplementary file 6 — Supplementary material 6—Curves of m/z48.052 (C13CH6OH+ - tentatively identified as an isotope of ethanol) of yeast strains, medium and blank samples. Curves of each sample represent mean value and standard error of each sample type for each time point upon smoothing. Grey rectangle shows the period when samples were measured with fastGC PTR-ToF-MS. This figure corresponds to Figure 2B (PNG 273 KB) [file 11306_2017_1259_MOESM6_ESM.png]

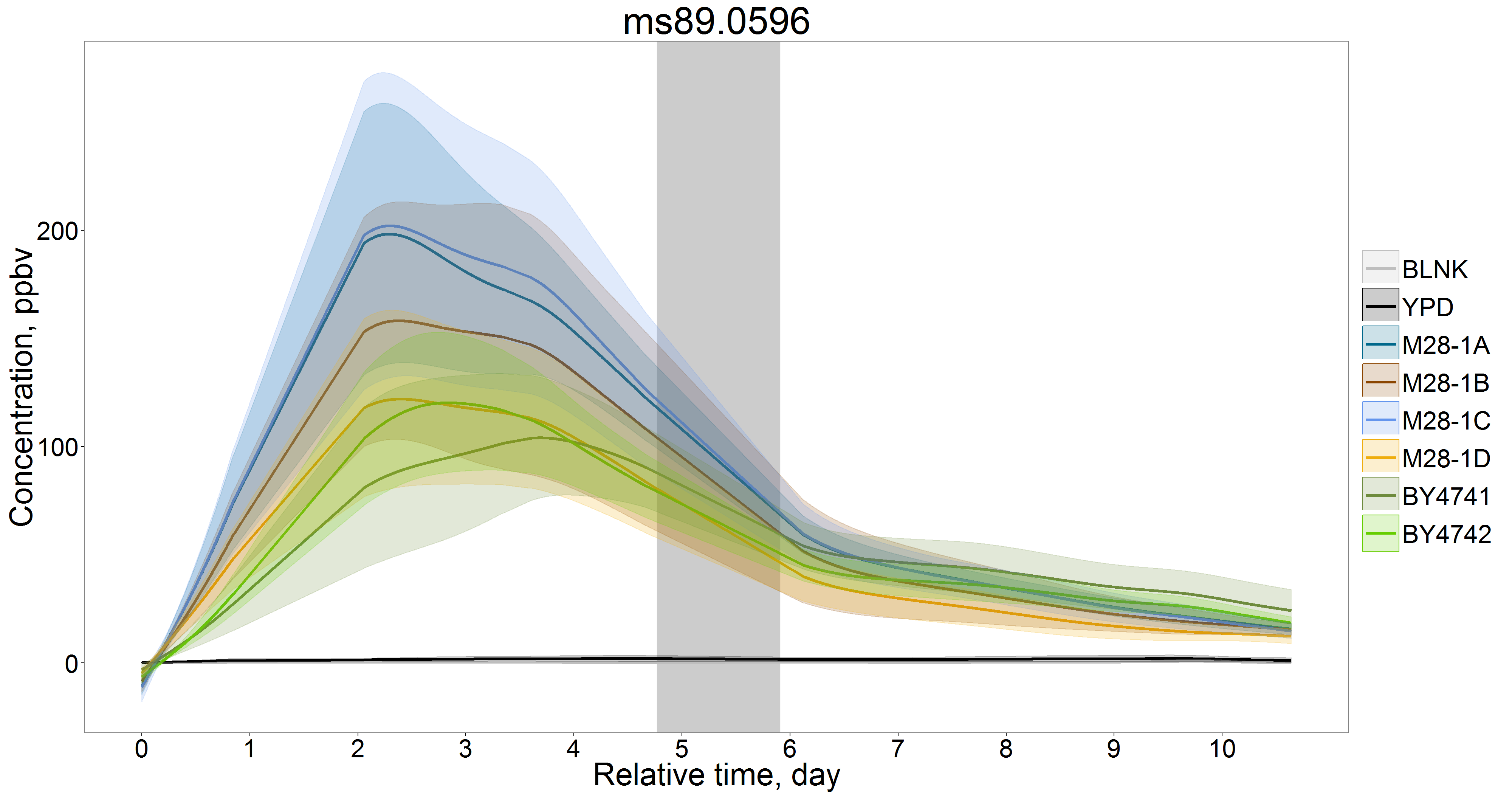

Supplement: Supplementary file 7 — Supplementary Figure 7—Curves of m/z89.060 (C4H8O2H+ - tentatively identified as ethyl acetate, isobutyric acid, butanoic acid, and acetoin) of yeast strains, medium and blank samples. Curves of each sample represent mean value and standard error of each sample type for each time point upon smoothing. Grey rectangle shows the period when samples were measured with fastGC PTR-ToF-MS. This figure corresponds to Figure 2C (PNG 229 KB) [file 11306_2017_1259_MOESM7_ESM.png]

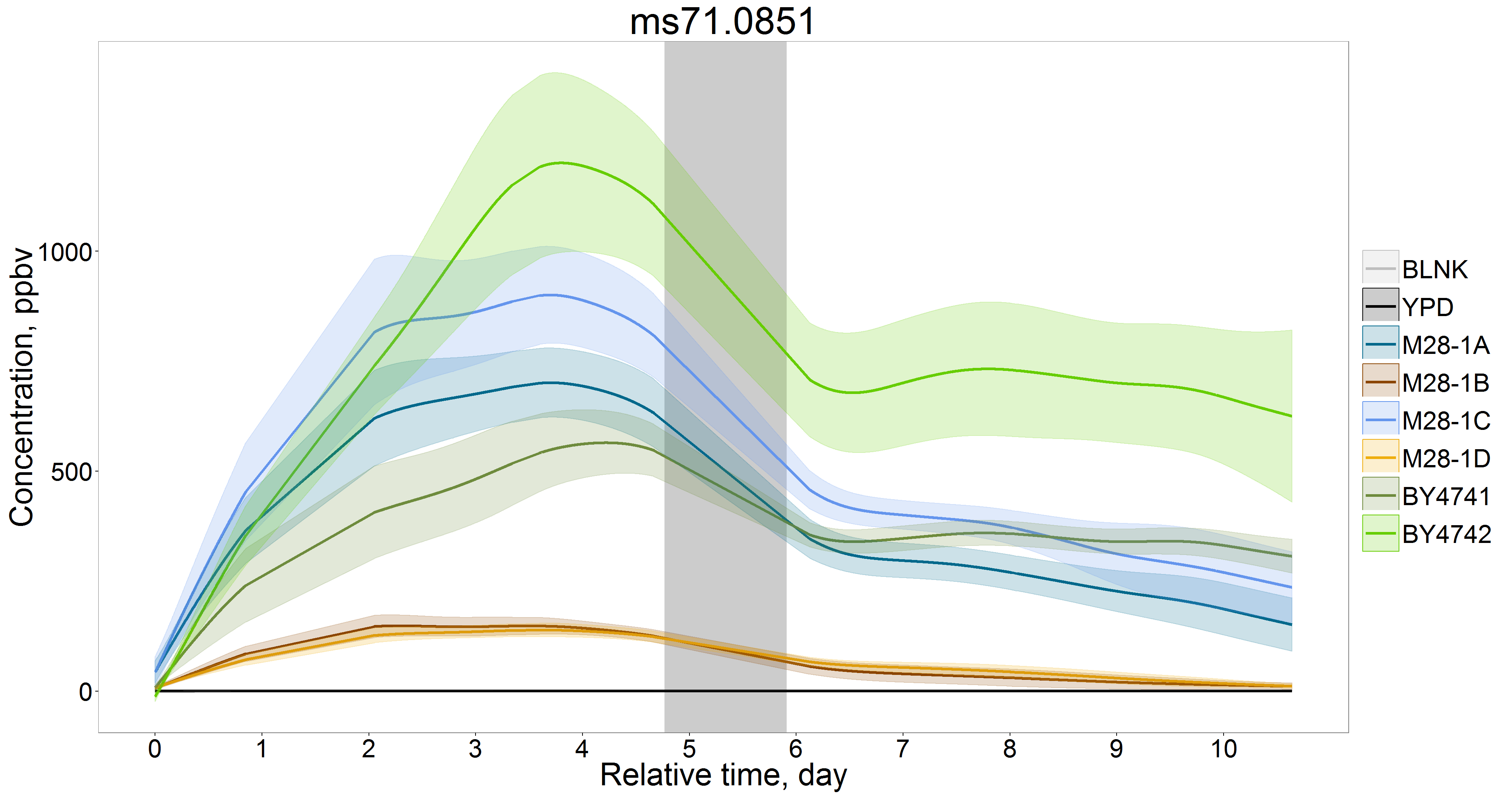

Supplement: Supplementary file 8 — Supplementary Figure 8—Curves of m/z71.085 (C5H11 + - tentatively identified as a fragment of alcohols such as 3-methyl-1-butanol and 2-methyl-1-butanol) of yeast strains, medium and blank samples. Curves of each sample represent mean value and standard error of each sample type for each time point upon smoothing. Grey rectangle shows the period when samples were measured with fastGC PTR-ToF-MS. This figure corresponds to Figure 2D (PNG 181 KB) [file 11306_2017_1259_MOESM8_ESM.png]

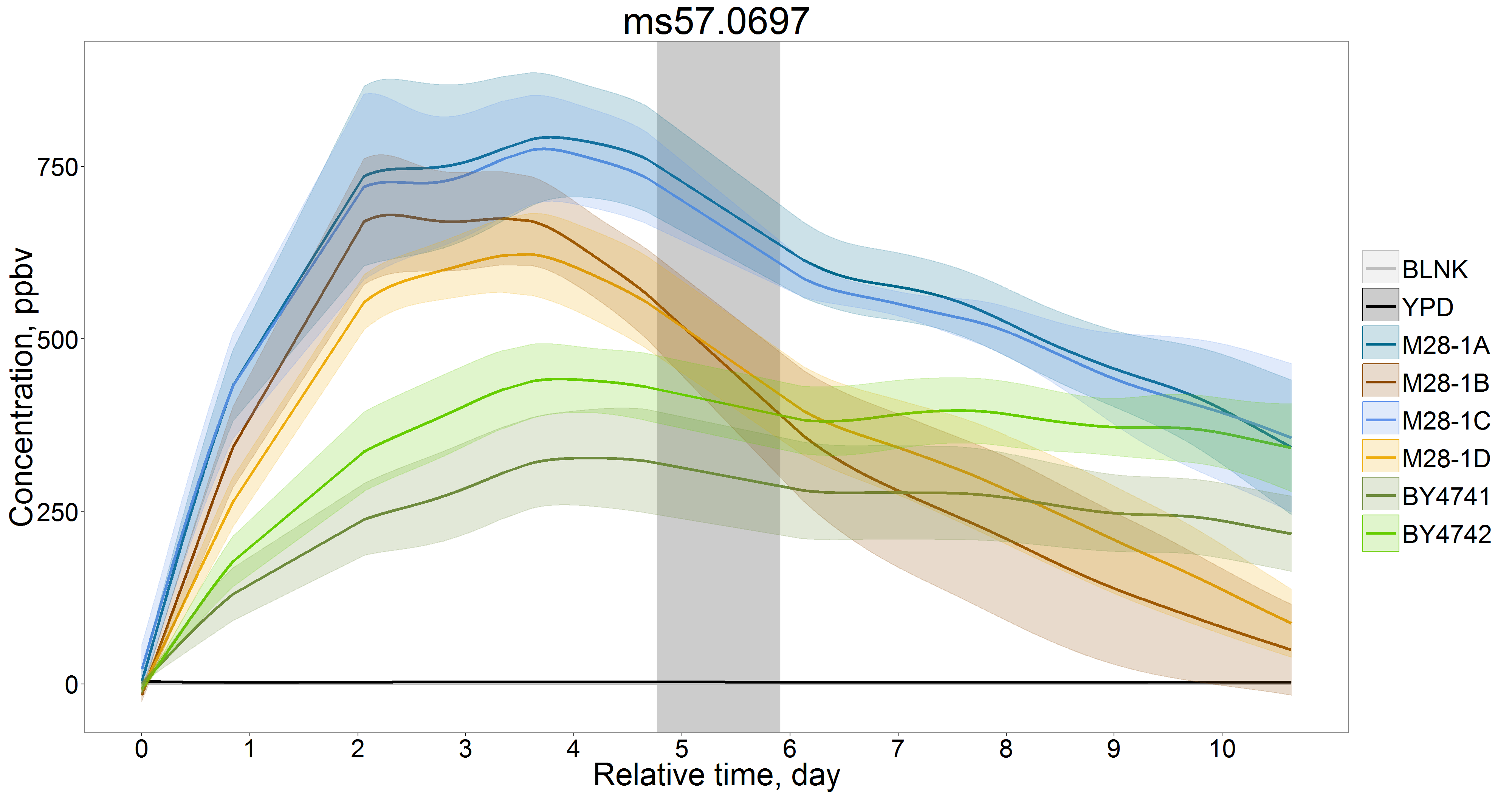

Supplement: Supplementary file 9 — Supplementary Figure 9—Curves of m/z57.069 (C4H9 + - tentatively identified as a fragment of butanol isomers) of yeast strains, medium and blank samples. Curves of each sample represent mean value and standard error of each sample type for each time point upon smoothing. Grey rectangle shows the period when samples were measured with fastGC PTR-ToF-MS. This figure corresponds to Figure 2E (PNG 234 KB) [file 11306_2017_1259_MOESM9_ESM.png]

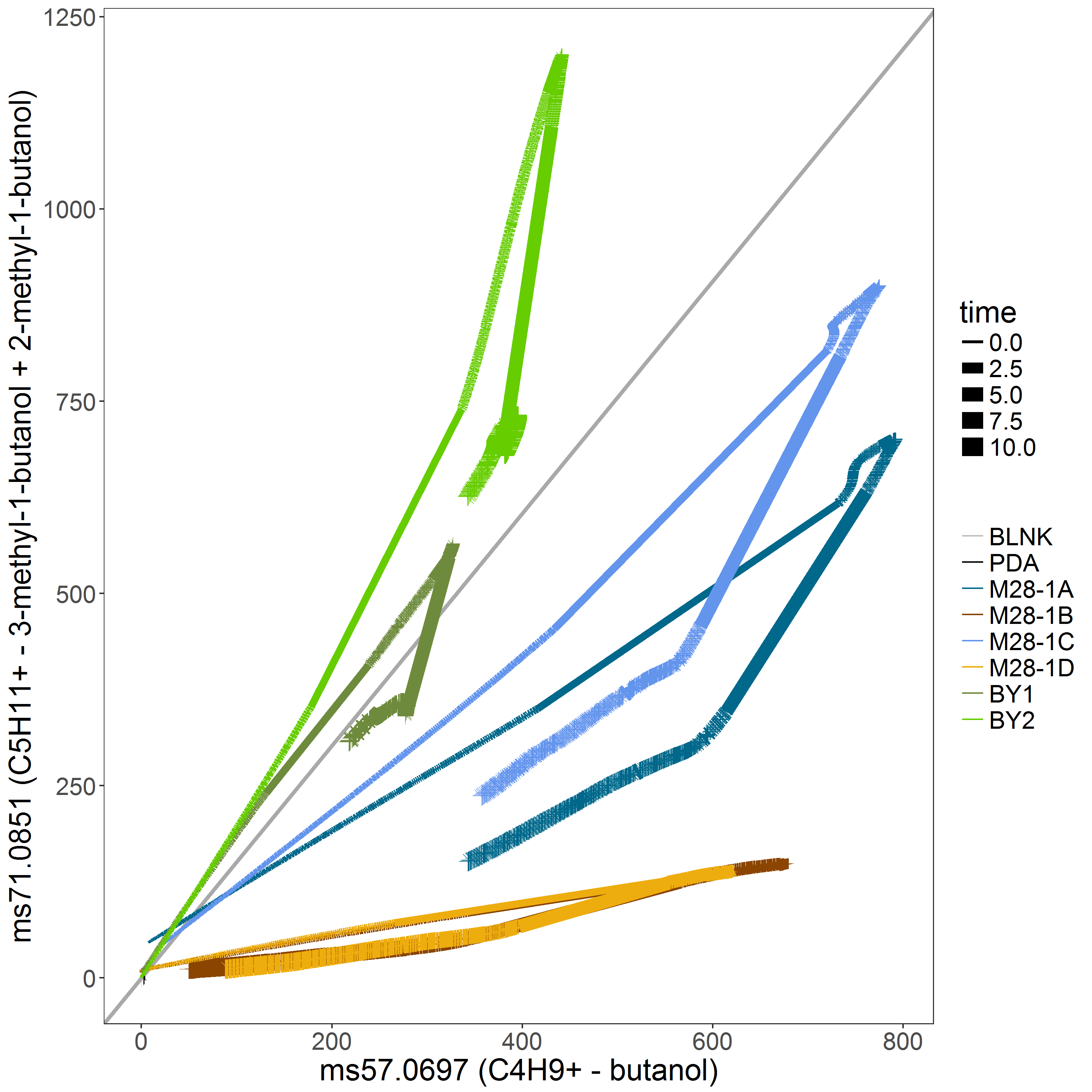

Supplement: Supplementary file 10 — Supplementary Figure 10—Scatter plot of m/z57.069 (C4H9 + - tentatively identified as a dehydrated fragment of butanol isomers) and m/z71.085 (C5H11 + - tentatively identified as a dehydrated fragment of alcohols such as 3-methyl-1-butanol and 2-methyl-1-butanol) of yeast strains, medium and blank samples. Curves of each sample represent mean value of each sample type for each time point upon smoothing. The gap in the lines corresponds to the measurement by fastGC PTR-ToF-MS. The inclination of the curves compares the emission of these two mass peaks (PNG 127 KB) [file 11306_2017_1259_MOESM10_ESM.png]

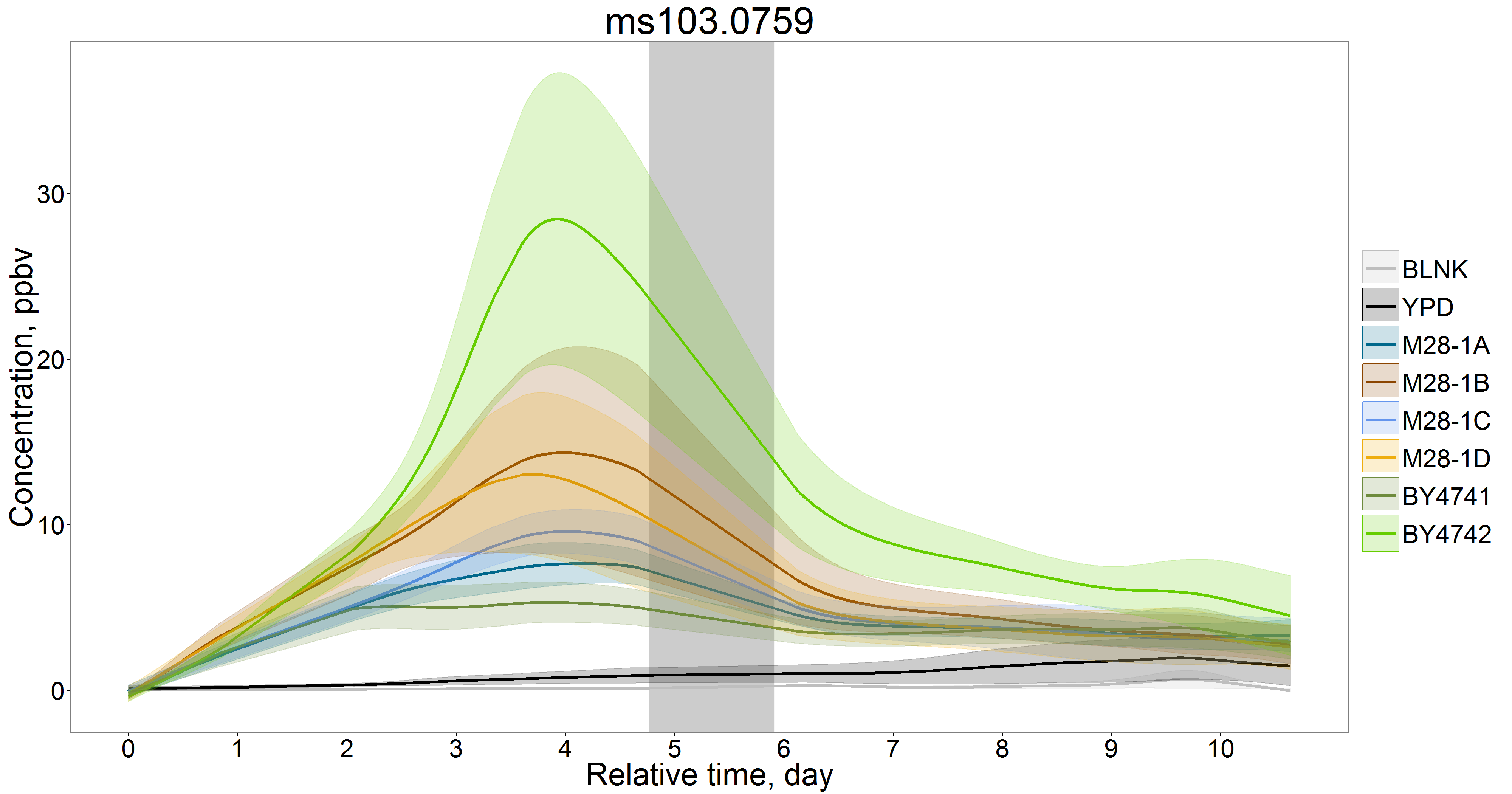

Supplement: Supplementary file 11 — Supplementary Figure 11—Curves of m/z103.076 (C5H10O2H+ - tentatively identified as isovaleric acid, ethyl propanoate, 2-methylbutanoic acid) of yeast strains, medium and blank samples. Curves of each sample represent mean value and standard error of each sample type for each time point upon smoothing. Grey rectangle shows the period when samples were measured with fastGC PTR-ToF-MS. This figure corresponds to Figure 2F (PNG 172 KB) [file 11306_2017_1259_MOESM11_ESM.png]

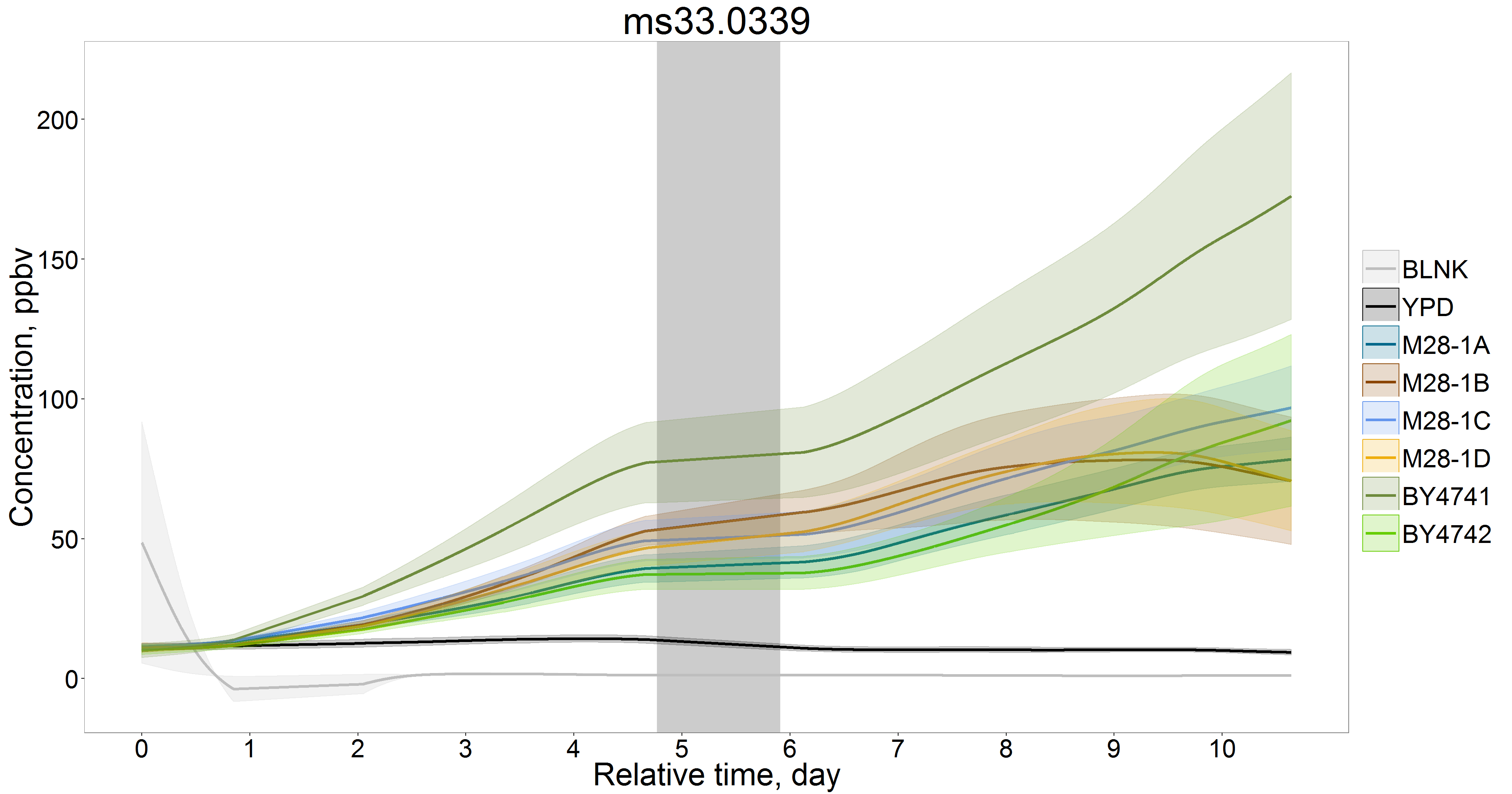

Supplement: Supplementary file 12 — Supplementary Figure 12—Curves of m/z33.034 (CH4OH+ - tentatively identified as methanol) of yeast strains, medium and blank samples. Curves of each sample represent mean value and standard error of each sample type for each time point upon smoothing. Grey rectangle shows the period when samples were measured with fastGC PTR-ToF-MS. This figure corresponds to Figure 2G (PNG 150 KB) [file 11306_2017_1259_MOESM12_ESM.png]

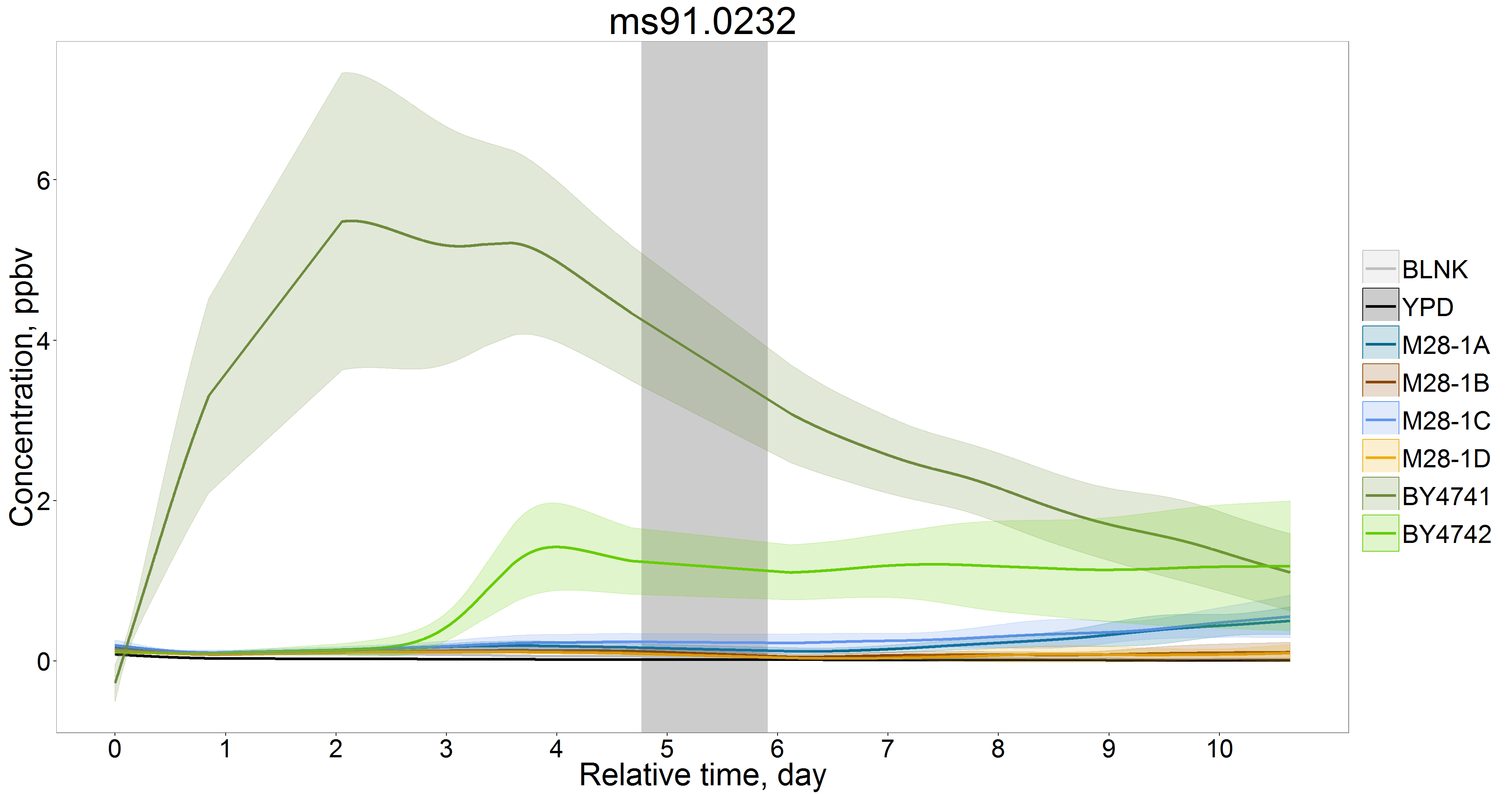

Supplement: Supplementary file 13 — Supplementary Figure 13—Curves of m/z91.023 (C3H6OSH+ - tentatively identified as S-methyl thioacetate) of yeast strains, medium and blank samples. Curves of each sample represent mean value and standard error of each sample type for each time point upon smoothing. Grey rectangle shows the period when samples were measured with fastGC PTR-ToF-MS. This figure corresponds to Figure 2H (PNG 126 KB) [file 11306_2017_1259_MOESM13_ESM.png]

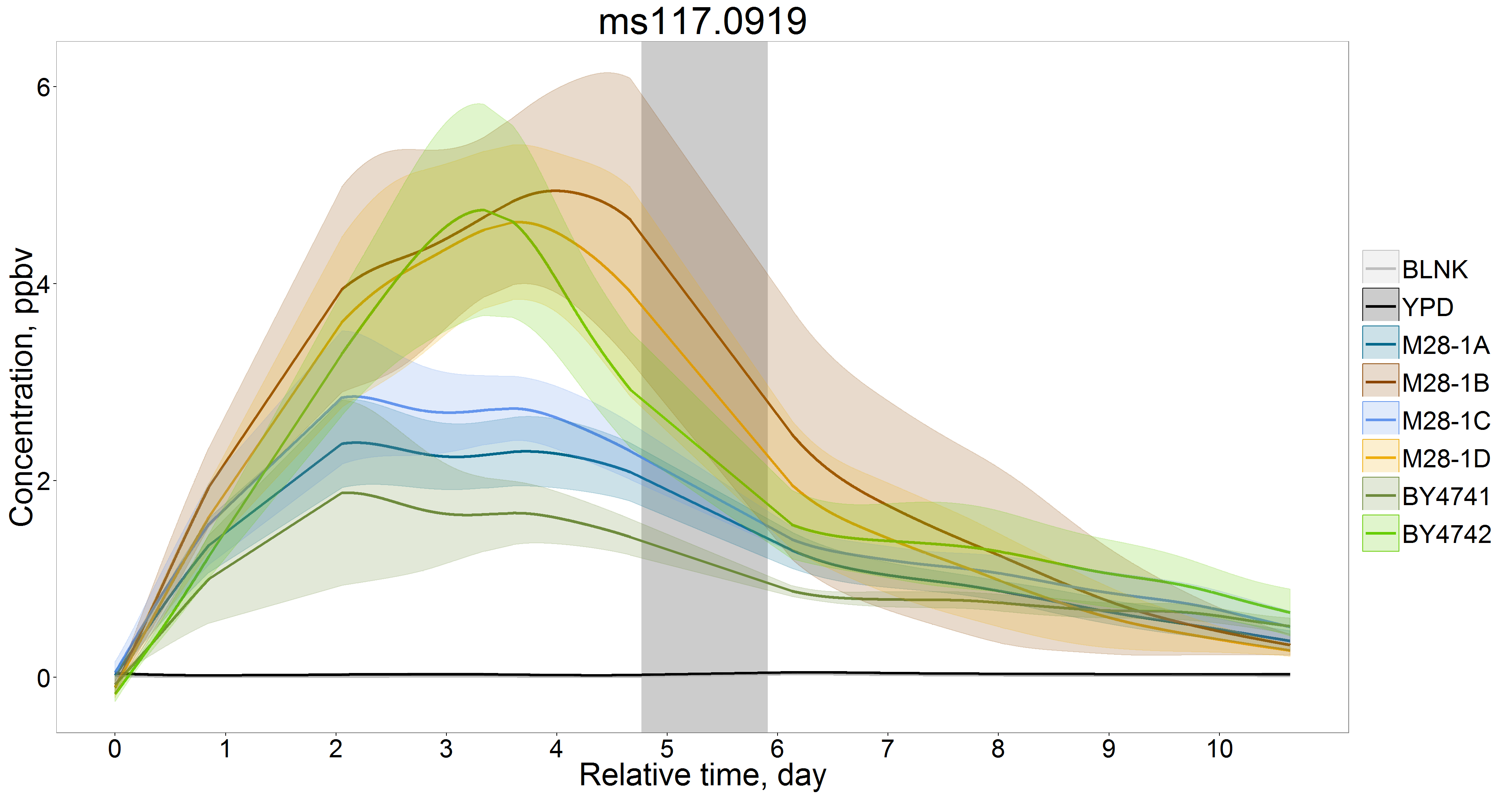

Supplement: Supplementary file 14 — Supplementary Figure 14—Curves of m/z117.092 (C6H12O2H+ - tentatively identified as ethyl butyrate, ethyl isobutyrate, isobutyl acetate) of yeast strains, medium and blank samples. Curves of each sample represent mean value and standard error of each sample type for each time point upon smoothing. Grey rectangle shows the period when samples were measured with fastGC PTR-ToF-MS. This figure corresponds to Figure 2I (PNG 236 KB) [file 11306_2017_1259_MOESM14_ESM.png]
