# Supplementary material for: Non-invasive real time monitoring of yeast volatilome by PTR-ToF-MS
Source: Metabolomics. 2017 Aug 31;13(10):118. doi: 10.1007/s11306-017-1259-y (PMC5579147; doi:10.1007/s11306-017-1259-y)
Supplement: Supplementary file 15 — Supplementary Table 1—VOC significantly different from blank samples and also significantly different either between M28 segregants or between BY4741 and BY4742. Symbol ‘*’ represts compounds found during measurements of one sample from each yeast strain with GC-MS (DOCX 74 KB) [file 11306_2017_1259_MOESM15_ESM.docx]

| ***Supplementary table I.*** *VOC significantly different from blank samples and also significantly different either between M28 segregants or between BY4741 and BY4742. Symbol ‘*’ represents compounds found during measurements of one sample from each yeast strain with GC-MS* | | | | | | | | | | | | | | | | | | | | | | | | | | | | | | | | | |  | |
| --- | --- | --- | --- | --- | --- | --- | --- | --- | --- | --- | --- | --- | --- | --- | --- | --- | --- | --- | --- | --- | --- | --- | --- | --- | --- | --- | --- | --- | --- | --- | --- | --- | --- | --- | --- |
| Measured mass | Theoretical mass | Sum Formula | Concentration, ppbv | | | | | | | | | | | | | | | | | | | p-value | Concentration, ppbv | | | | | | | | | p-value | Tentative identification |  | |
|  |  |  | M28-1A | | | | M28-1B | | | | | | M28-1C | | | | M28-1D | | | | |  | BY4741 | | | | | BY4742 | | | |  |  |  | |
| 26.016 | 26.0151 | C_2_H_2_^+^ | 1.19 | ± | 0.16 | *a* | | 1.16 | ± | 0.15 | *a* | 1.24 | | ± | 0.23 | *a* | | 1.12 | ± | 0.09 | *a* | 3.14E-01 | 0.81 | ± | 0.10 | *b* | 1.01 | | ± | 0.14 | *a* | 9.88E-04 |  |  |  |
| 26.035 | n.a. | unidentified | 0.17 | ± | 0.05 | *ab* | | 0.11 | ± | 0.04 | *b* | 0.19 | | ± | 0.03 | *a* | | 0.14 | ± | 0.04 | *ab* | 1.30E-04 | 0.22 | ± | 0.06 | *a* | 0.24 | | ± | 0.05 | *a* | 3.35E-01 |  |  |  |
| 27.025 | 27.0229 | C_2_H_3_^+^ | 22.63 | ± | 1.36 | *a* | | 12.26 | ± | 3.29 | *b* | 24.62 | | ± | 1.29 | *a* | | 14.03 | ± | 2.31 | *b* | 5.43E-19 | 27.24 | ± | 1.37 | *b* | 33.01 | | ± | 1.97 | *a* | 2.89E-07 | Common fragment |  |  |
| 28.031 | 28.0307 | C_2_H_4_^+^ | 1.54 | ± | 0.13 | *a* | | 1.60 | ± | 0.17 | *a* | 1.57 | | ± | 0.15 | *a* | | 1.54 | ± | 0.10 | *a* | 7.01E-01 | 1.17 | ± | 0.11 | *b* | 1.37 | | ± | 0.12 | *a* | 7.31E-04 | Common fragment |  |  |
| 31.018 | 31.0178 | CH_2_OH^+^ | 19.11 | ± | 2.36 | *a* | | 22.01 | ± | 2.62 | *a* | 19.02 | | ± | 2.20 | *a* | | 21.17 | ± | 2.32 | *a* | 6.03E-03 | 12.39 | ± | 1.44 | *a* | 13.31 | | ± | 1.44 | *a* | 1.63E-01 |  |  |  |
| 31.045 | n.a. | unidentified | 0.39 | ± | 0.07 | *a* | | 0.31 | ± | 0.08 | *a* | 0.38 | | ± | 0.09 | *a* | | 0.32 | ± | 0.08 | *a* | 2.32E-02 | 0.49 | ± | 0.08 | *a* | 0.55 | | ± | 0.10 | *a* | 1.28E-01 |  |  |  |
| 33.034 | 33.0335 | CH_4_OH^+^ | 39.76 | ± | 4.85 | *b* | | 53.43 | ± | 5.09 | *a* | 49.86 | | ± | 7.34 | *a* | | 47.19 | ± | 6.49 | *ab* | 1.54E-05 | 78.28 | ± | 14.39 | *a* | 37.84 | | ± | 5.54 | *b* | 5.05E-08 | Methanol |  |  |
| 34.995 | 34.9949 | H_2_SH^+^ | 0.69 | ± | 0.40 | *b* | | 0.21 | ± | 0.22 | *b* | 2.09 | | ± | 0.96 | *a* | | 0.20 | ± | 0.20 | *b* | 3.25E-11 | 4.33 | ± | 1.45 | *a* | 4.49 | | ± | 2.04 | *a* | 8.37E-01 | Hydrogen sulfide |  |  |
| 38.019 | n.a. | unidentified | 0.41 | ± | 0.08 | *a* | | 0.21 | ± | 0.05 | *b* | 0.46 | | ± | 0.09 | *a* | | 0.19 | ± | 0.07 | *b* | 3.14E-13 | 0.31 | ± | 0.06 | *b* | 0.44 | | ± | 0.08 | *a* | 5.34E-04 |  |  |  |
| 38.034 | n.a. | unidentified | 5.99 | ± | 0.32 | *a* | | 5.39 | ± | 0.45 | *b* | 6.06 | | ± | 0.24 | *a* | | 5.54 | ± | 0.27 | *ab* | 7.43E-06 | 6.55 | ± | 0.26 | *a* | 6.56 | | ± | 0.26 | *a* | 9.62E-01 |  |  |  |
| 39.023 | 39.0229 | C_3_H_3_^+^ | 1503.22 | ± | 159.30 | *b* | | 591.44 | ± | 29.37 | *c* | 1764.16 | | ± | 209.20 | *a* | | 571.77 | ± | 39.26 | *c* | 7.82E-28 | 982.57 | ± | 125.73 | *b* | 1800.10 | | ± | 240.55 | *a* | 1.00E-08 | Common fragment | [5] |  |
| 41.038 | 41.0385 | C_3_H_5_^+^ | 1469.03 | ± | 164.55 | *b* | | 581.83 | ± | 31.84 | *c* | 1728.53 | | ± | 216.88 | *a* | | 556.16 | ± | 39.83 | *c* | 7.88E-27 | 941.40 | ± | 123.64 | *b* | 1698.57 | | ± | 228.69 | *a* | 1.69E-08 | Ester fragment | [6] |  |
| 42.010 | 42.0100 | C_2_H_2_O^+^ | 4.92 | ± | 0.18 | *a* | | 3.96 | ± | 0.51 | *b* | 5.00 | | ± | 0.15 | *a* | | 4.34 | ± | 0.31 | *b* | 2.38E-10 | 4.81 | ± | 0.19 | *b* | 5.28 | | ± | 0.14 | *a* | 3.09E-06 |  |  |  |
| 43.017 | 43.0178 | C_2_H_3_O^+^ | 972.68 | ± | 191.48 | *a* | | 790.92 | ± | 338.18 | *a* | 1003.08 | | ± | 312.94 | *a* | | 607.73 | ± | 174.62 | *a* | 2.07E-03 | 672.56 | ± | 141.85 | *a* | 608.33 | | ± | 119.04 | *a* | 2.73E-01 | Common fragment | [6] |  |
| 43.054 | 43.0542 | C_3_H_7_^+^ | 728.95 | ± | 80.41 | *b* | | 214.14 | ± | 23.09 | *c* | 896.06 | | ± | 95.36 | *a* | | 216.71 | ± | 20.14 | *c* | 6.02E-31 | 594.26 | ± | 70.15 | *b* | 1074.90 | | ± | 136.70 | *a* | 5.49E-09 | Common fragment | [5] |  |
| 45.993 | 46.0004 | ^13^CO_2_H^+^ | 0.87 | ± | 0.06 | *a* | | 0.90 | ± | 0.06 | *a* | 0.86 | | ± | 0.06 | *a* | | 0.91 | ± | 0.05 | *a* | 1.87E-01 | 0.85 | ± | 0.06 | *a* | 0.86 | | ± | 0.07 | *a* | 6.78E-01 | Carbon dioxide |  |  |
| 46.037 | 46.0368 | C^13^CH_4_OH^+^ | 81.02 | ± | 17.11 | *ab* | | 81.68 | ± | 15.01 | *ab* | 50.80 | | ± | 10.49 | *b* | | 103.66 | ± | 28.31 | *a* | 4.01E-07 | 41.06 | ± | 6.14 | *a* | 42.17 | | ± | 44.43 | *a* | 9.39E-01 | Acetaldehyde (isotope) |  |  |
| 48.052 | 48.0524 | C^13^CH_6_OH^+^ | 279.68 | ± | 38.91 | *a* | | 199.43 | ± | 64.32 | *b* | 276.00 | | ± | 36.58 | *ab* | | 232.14 | ± | 43.44 | *ab* | 2.48E-04 | 404.43 | ± | 32.70 | *a* | 391.99 | | ± | 27.14 | *a* | 3.53E-01 | Ethanol (isotope) |  |  |
| 49.011 | 49.0106 | CH_4_SH^+^ | 0.54 | ± | 0.10 | *b* | | 0.51 | ± | 0.08 | *b* | 0.81 | | ± | 0.18 | *a* | | 0.46 | ± | 0.05 | *b* | 9.51E-09 | 5.40 | ± | 4.06 | *a* | 4.99 | | ± | 1.96 | *a* | 7.67E-01 | Methanethiol | [7] |  |
| 53.039 | 53.0385 | C_4_H_5_^+^ | 2.88 | ± | 0.33 | *a* | | 1.31 | ± | 0.13 | *b* | 3.26 | | ± | 0.34 | *a* | | 1.36 | ± | 0.16 | *b* | 6.74E-25 | 2.21 | ± | 0.23 | *b* | 3.60 | | ± | 0.40 | *a* | 1.10E-08 |  |  |  |
| 54.008 | n.a. | unidentified | 0.13 | ± | 0.03 | *a* | | 0.07 | ± | 0.01 | *b* | 0.14 | | ± | 0.03 | *a* | | 0.05 | ± | 0.02 | *b* | 5.37E-12 | 0.10 | ± | 0.03 | *a* | 0.13 | | ± | 0.03 | *a* | 1.40E-02 |  |  |  |
| 55.018 | n.a. | unidentified | 0.95 | ± | 0.09 | *a* | | 0.45 | ± | 0.10 | *b* | 1.02 | | ± | 0.10 | *a* | | 0.40 | ± | 0.05 | *b* | 3.86E-24 | 0.74 | ± | 0.14 | *b* | 1.20 | | ± | 0.13 | *a* | 2.70E-07 |  |  |  |
| 55.055 | 55.0542 | C_4_H_7_^+^ | 25.87 | ± | 2.69 | *a* | | 11.88 | ± | 0.93 | *b* | 29.26 | | ± | 3.20 | *a* | | 11.75 | ± | 1.06 | *b* | 4.92E-26 | 18.94 | ± | 1.68 | *b* | 32.42 | | ± | 3.69 | *a* | 2.10E-09 | Common fragment |  |  |
| 57.034 | 57.0335 | C_3_H_4_OH^+^ | 37.23 | ± | 3.39 | *a* | | 20.57 | ± | 8.37 | *b* | 43.51 | | ± | 4.10 | *a* | | 19.69 | ± | 5.78 | *b* | 1.55E-14 | 33.90 | ± | 3.53 | *b* | 70.42 | | ± | 12.87 | *a* | 5.02E-08 | Common fragment | [5] |  |
| 57.069 | 57.0698 | C_4_H_9_^+^ | 778.02 | ± | 82.96 | *a* | | 590.04 | ± | 63.38 | *b* | 749.93 | | ± | 72.68 | *a* | | 569.45 | ± | 64.03 | *b* | 4.05E-10 | 328.46 | ± | 75.06 | *b* | 438.31 | | ± | 53.15 | *a* | 9.62E-04 | Isobutyl alcohol*, 1-butanol*,  high alcohol fragment | [1, 4] |  |
| 58.982 | n.a. | unidentified | 0.53 | ± | 0.09 | *a* | | 0.43 | ± | 0.06 | *a* | 0.52 | | ± | 0.08 | *a* | | 0.47 | ± | 0.07 | *a* | 4.30E-03 | 0.66 | ± | 0.07 | *a* | 0.61 | | ± | 0.05 | *a* | 4.33E-02 |  |  |  |
| 59.049 | 59.0491 | C_3_H_0_OH^+^ | 145.20 | ± | 14.93 | *a* | | 150.72 | ± | 19.99 | *a* | 149.34 | | ± | 16.16 | *a* | | 134.92 | ± | 14.93 | *a* | 1.00E-01 | 141.87 | ± | 10.90 | *a* | 170.80 | | ± | 28.17 | *a* | 6.71E-03 | Acetone* | [6] |  |
| 61.028 | 61.0284 | C_2_H_4_O_2_H^+^ | 539.68 | ± | 122.76 | *a* | | 466.44 | ± | 197.05 | *a* | 560.97 | | ± | 184.09 | *a* | | 342.02 | ± | 106.65 | *a* | 6.04E-03 | 323.42 | ± | 72.41 | *a* | 293.77 | | ± | 60.00 | *a* | 3.18E-01 | Acetic acid*, common fragment | [1] |  |
| 63.008 | n.a. | unidentified | 0.70 | ± | 0.11 | *a* | | 0.69 | ± | 0.15 | *a* | 0.68 | | ± | 0.15 | *a* | | 0.60 | ± | 0.13 | *a* | 3.20E-01 | 0.66 | ± | 0.09 | *a* | 0.40 | | ± | 0.10 | *b* | 5.89E-06 |  |  |  |
| 64.035 | n.a. | unidentified | 0.07 | ± | 0.03 | *a* | | 0.07 | ± | 0.04 | *a* | 0.09 | | ± | 0.03 | *a* | | 0.05 | ± | 0.03 | *a* | 6.52E-02 | 0.05 | ± | 0.02 | *a* | 0.05 | | ± | 0.02 | *a* | 5.66E-01 |  |  |  |
| 67.055 | 67.0542 | C_5_H_7_^+^ | 5.00 | ± | 0.48 | *a* | | 1.83 | ± | 0.44 | *b* | 5.50 | | ± | 0.57 | *a* | | 1.94 | ± | 0.32 | *b* | 3.11E-26 | 4.68 | ± | 0.46 | *b* | 7.42 | | ± | 0.89 | *a* | 4.37E-08 |  |  |  |
| 68.053 | 68.0531 | unidentified | 0.48 | ± | 0.05 | *a* | | 0.33 | ± | 0.06 | *b* | 0.51 | | ± | 0.07 | *a* | | 0.32 | ± | 0.06 | *b* | 9.61E-11 | 0.47 | ± | 0.06 | *a* | 0.57 | | ± | 0.07 | *a* | 2.61E-03 |  |  |  |
| 69.070 | 69.0699 | C_5_H_9_^+^ | 22.05 | ± | 3.21 | *a* | | 7.83 | ± | 0.76 | *b* | 19.90 | | ± | 3.19 | *a* | | 7.74 | ± | 1.13 | *b* | 1.22E-21 | 12.02 | ± | 1.35 | *b* | 16.60 | | ± | 3.32 | *a* | 6.56E-04 | Common fragment |  |  |
| 71.048 | 71.0491 | C_4_H_6_OH^+^ | 2.63 | ± | 0.36 | *b* | | 3.54 | ± | 0.60 | *a* | 2.53 | | ± | 0.41 | *b* | | 3.07 | ± | 0.44 | *ab* | 6.72E-06 | 4.15 | ± | 6.46 | *a* | 3.26 | | ± | 1.16 | *a* | 6.59E-01 | Butenal | [6] |  |
| 71.085 | 71.0855 | C_5_H_11_^+^ | 662.86 | ± | 80.36 | *b* | | 133.33 | ± | 14.72 | *c* | 846.00 | | ± | 98.93 | *a* | | 129.95 | ± | 12.33 | *c* | 1.08E-31 | 570.80 | ± | 60.34 | *b* | 1157.57 | | ± | 178.23 | *a* | 6.30E-09 | 3-methyl-1-butanol + 2-methyl-1-butanol*; 2-pentanol* | [1, 4] |  |
| 73.065 | 73.0648 | C_4_H_8_OH^+^ | 43.05 | ± | 6.95 | *a* | | 35.61 | ± | 2.92 | *a* | 37.34 | | ± | 5.36 | *a* | | 37.99 | ± | 5.33 | *a* | 9.53E-03 | 25.25 | ± | 2.67 | *a* | 33.84 | | ± | 12.08 | *a* | 4.09E-02 | Butanal | [1, 6] |  |
| 75.044 | 75.0441 | C_3_H_6_O_2_H^+^ | 25.87 | ± | 4.66 | *a* | | 52.58 | ± | 27.08 | *a* | 32.42 | | ± | 5.26 | *a* | | 41.48 | ± | 19.29 | *a* | 2.46E-03 | 17.14 | ± | 1.83 | *b* | 83.04 | | ± | 27.68 | *a* | 4.35E-07 | Propanoic acid*, Fragment of m/z 103.076 | [1, 6] |  |
| 79.039 | 79.0390 | C_2_H_6_O_3_H^+^ | 0.52 | ± | 0.12 | *a* | | 0.44 | ± | 0.15 | *a* | 0.53 | | ± | 0.17 | *a* | | 0.30 | ± | 0.12 | *a* | 8.75E-04 | 0.33 | ± | 0.07 | *a* | 0.26 | | ± | 0.08 | *a* | 4.46E-02 |  |  |  |
| 81.038 | n.a. | unidentified | 0.77 | ± | 0.07 | *a* | | 0.69 | ± | 0.10 | *a* | 0.76 | | ± | 0.05 | *a* | | 0.66 | ± | 0.10 | *a* | 5.29E-03 | 0.79 | ± | 0.12 | *a* | 0.79 | | ± | 0.06 | *a* | 8.64E-01 |  |  |  |
| 81.069 | 81.0699 | C_6_H_9_^+^ | 0.23 | ± | 0.04 | *ab* | | 0.17 | ± | 0.04 | *b* | 0.27 | | ± | 0.05 | *a* | | 0.17 | ± | 0.03 | *b* | 1.83E-07 | 0.20 | ± | 0.03 | *b* | 0.27 | | ± | 0.05 | *a* | 6.61E-04 |  |  |  |
| 82.069 | n.a. | unidentified | 0.03 | ± | 0.01 | *a* | | 0.03 | ± | 0.01 | *a* | 0.03 | | ± | 0.02 | *a* | | 0.03 | ± | 0.01 | *a* | 9.53E-01 | 0.04 | ± | 0.02 | *a* | 0.04 | | ± | 0.01 | *a* | 3.99E-01 |  |  |  |
| 83.049 | 83.0491 | C_5_H_6_OH^+^ | 0.18 | ± | 0.03 | *ab* | | 0.22 | ± | 0.06 | *a* | 0.16 | | ± | 0.02 | *b* | | 0.23 | ± | 0.03 | *a* | 8.79E-05 | 0.16 | ± | 0.04 | *a* | 0.23 | | ± | 0.04 | *a* | 3.06E-03 |  |  |  |
| 83.086 | 83.0855 | C_6_H_11_^+^ | 0.22 | ± | 0.04 | *a* | | 0.31 | ± | 0.08 | *a* | 0.24 | | ± | 0.03 | *a* | | 0.27 | ± | 0.06 | *a* | 1.16E-03 | 0.13 | ± | 0.02 | *b* | 0.17 | | ± | 0.02 | *a* | 1.39E-04 |  |  |  |
| 85.065 | 85.0648 | C_5_H_8_OH^+^ | 0.43 | ± | 0.07 | *a* | | 0.34 | ± | 0.05 | *a* | 0.42 | | ± | 0.07 | *a* | | 0.33 | ± | 0.05 | *a* | 2.36E-04 | 0.44 | ± | 0.20 | *a* | 0.49 | | ± | 0.09 | *a* | 4.20E-01 |  |  |  |
| 87.081 | 87.0804 | C_5_H_10_OH^+^ | 30.42 | ± | 5.92 | *a* | | 19.37 | ± | 5.64 | *bc* | 27.32 | | ± | 5.48 | *ab* | | 16.36 | ± | 5.71 | *c* | 3.30E-07 | 10.52 | ± | 1.73 | *b* | 21.84 | | ± | 5.16 | *a* | 2.59E-06 | 2-pentanone*, 3-methyl-3-buten-1-ol*, 3-methyl butanal* | [1] |  |
| 89.060 | 89.0597 | C_4_H_8_O_2_H^+^ | 128.58 | ± | 22.55 | *a* | | 114.75 | ± | 47.56 | *a* | 132.14 | | ± | 38.60 | *a* | | 87.78 | ± | 25.78 | *a* | 1.40E-02 | 93.77 | ± | 18.79 | *a* | 86.36 | | ± | 16.89 | *a* | 3.53E-01 | Ethyl acetate*, Isobutyric acid*, Acetoin*, Butanoic acid* | [1] |  |
| 91.023 | 91.0212 | C_3_H_6_OSH^+^ | 0.18 | ± | 0.05 | *ab* | | 0.14 | ± | 0.05 | *b* | 0.25 | | ± | 0.11 | *a* | | 0.10 | ± | 0.02 | *b* | 7.73E-06 | 4.49 | ± | 0.90 | *a* | 1.24 | | ± | 0.42 | *b* | 1.49E-09 | S-Methyl thioacetate | [7] |  |
| 91.073 | 91.0753 | C_4_H_10_O_2_H^+^ | 2.85 | ± | 0.62 | *a* | | 2.16 | ± | 0.70 | *a* | 2.04 | | ± | 0.43 | *a* | | 2.74 | ± | 0.81 | *a* | 6.45E-03 | 2.18 | ± | 0.34 | *a* | 1.97 | | ± | 1.24 | *a* | 6.21E-01 | Butanediol |  |  |
| 92.064 | n.a. | unidentified | 0.11 | ± | 0.03 | *a* | | 0.12 | ± | 0.05 | *a* | 0.14 | | ± | 0.04 | *a* | | 0.13 | ± | 0.03 | *a* | 1.98E-01 | 0.10 | ± | 0.04 | *a* | 0.11 | | ± | 0.05 | *a* | 5.50E-01 |  |  |  |
| 95.094 | n.a. | unidentified | 0.40 | ± | 0.08 | *a* | | 0.27 | ± | 0.11 | *a* | 0.40 | | ± | 0.06 | *a* | | 0.28 | ± | 0.08 | *a* | 1.13E-04 | 0.76 | ± | 0.15 | *a* | 0.75 | | ± | 0.12 | *a* | 9.24E-01 |  |  |  |
| 99.044 | 99.0440 | C_5_H_6_O_2_H^+^ | 0.17 | ± | 0.03 | *a* | | 0.09 | ± | 0.03 | *b* | 0.18 | | ± | 0.03 | *a* | | 0.08 | ± | 0.02 | *b* | 3.10E-12 | 0.12 | ± | 0.03 | *a* | 0.13 | | ± | 0.03 | *a* | 2.66E-01 |  |  |  |
| 99.081 | 99.0804 | C_6_H_10_O^+^ | 0.05 | ± | 0.01 | *a* | | 0.05 | ± | 0.01 | *a* | 0.05 | | ± | 0.01 | *a* | | 0.04 | ± | 0.02 | *a* | 3.05E-02 | 0.05 | ± | 0.02 | *a* | 0.06 | | ± | 0.02 | *a* | 1.00E-01 |  |  |  |
| 101.060 | 101.0597 | C_5_H_8_O_2_H^+^ | 0.16 | ± | 0.02 | *a* | | 0.13 | ± | 0.02 | *a* | 0.15 | | ± | 0.02 | *a* | | 0.14 | ± | 0.02 | *a* | 2.45E-02 | 0.13 | ± | 0.02 | *a* | 0.16 | | ± | 0.03 | *a* | 2.45E-02 |  |  |  |
| 103.045 | n.a. | unidentified | 0.13 | ± | 0.06 | *a* | | 0.10 | ± | 0.05 | *a* | 0.10 | | ± | 0.05 | *a* | | 0.10 | ± | 0.05 | *a* | 5.29E-01 | 0.14 | ± | 0.04 | *a* | 0.21 | | ± | 0.06 | *a* | 2.24E-03 |  |  |  |
| 103.076 | 103.0754 | C_5_H_10_O_2_H^+^ | 7.76 | ± | 1.03 | *a* | | 13.86 | ± | 6.64 | *a* | 9.39 | | ± | 1.43 | *a* | | 11.21 | ± | 4.87 | *a* | 6.76E-03 | 5.18 | ± | 1.05 | *b* | 25.30 | | ± | 8.13 | *a* | 2.65E-07 | Isovaleric acid*, Ethyl propanoate*, 2-methyl butanoic acid* | [1, 6] |  |
| 113.098 | 113.0961 | C_7_H_12_OH^+^ | 0.05 | ± | 0.01 | *a* | | 0.03 | ± | 0.01 | *a* | 0.05 | | ± | 0.02 | *a* | | 0.03 | ± | 0.01 | *a* | 2.31E-04 | 0.04 | ± | 0.02 | *a* | 0.07 | | ± | 0.02 | *a* | 1.49E-02 |  |  |  |
| 115.113 | 115.1117 | C_7_H_14_OH^+^ | 0.34 | ± | 0.08 | *a* | | 0.21 | ± | 0.08 | *ab* | 0.32 | | ± | 0.10 | *ab* | | 0.18 | ± | 0.11 | *b* | 7.01E-05 | 0.15 | ± | 0.04 | *b* | 0.30 | | ± | 0.08 | *a* | 2.20E-05 | 2-heptanone* |  |  |
| 117.039 | 117.0369 | unidentified | 0.16 | ± | 0.05 | *c* | | 0.46 | ± | 0.14 | *a* | 0.18 | | ± | 0.06 | *bc* | | 0.32 | ± | 0.09 | *ab* | 4.39E-10 | 0.05 | ± | 0.03 | *a* | 0.06 | | ± | 0.02 | *a* | 3.12E-01 |  |  |  |
| 117.092 | 117.0910 | C_6_H_12_O_2_H^+^ | 2.17 | ± | 0.30 | *b* | | 4.91 | ± | 1.44 | *a* | 2.38 | | ± | 0.29 | *b* | | 4.06 | ± | 1.11 | *a* | 1.89E-09 | 1.48 | ± | 0.21 | *b* | 3.14 | | ± | 0.62 | *a* | 1.50E-07 | Ethyl butyrate*, Ethyl isobutyrate*, Isobutyl acetate* | [1] |  |
| 118.980 | n.a. | unidentified | 0.10 | ± | 0.02 | *a* | | 0.09 | ± | 0.02 | *a* | 0.11 | | ± | 0.02 | *a* | | 0.10 | ± | 0.02 | *a* | 2.80E-01 | 0.12 | ± | 0.02 | *a* | 0.11 | | ± | 0.02 | *a* | 2.99E-01 |  |  |  |
| 119.091 | n.a. | unidentified | 0.14 | ± | 0.03 | *a* | | 0.13 | ± | 0.03 | *a* | 0.14 | | ± | 0.04 | *a* | | 0.14 | ± | 0.03 | *a* | 6.79E-01 | 0.16 | ± | 0.01 | *a* | 0.16 | | ± | 0.03 | *a* | 6.82E-01 |  |  |  |
| 121.068 | n.a. | unidentified | 0.24 | ± | 0.07 | *a* | | 0.14 | ± | 0.03 | *b* | 0.24 | | ± | 0.07 | *a* | | 0.15 | ± | 0.03 | *ab* | 1.06E-05 | 0.39 | ± | 0.11 | *a* | 0.34 | | ± | 0.06 | *a* | 1.80E-01 |  |  |  |
| 129.091 | 129.0910 | C_7_H_12_O_2_H^+^ | 0.12 | ± | 0.04 | *a* | | 0.06 | ± | 0.03 | *b* | 0.16 | | ± | 0.05 | *a* | | 0.04 | ± | 0.01 | *b* | 3.72E-11 | 0.07 | ± | 0.03 | *a* | 0.10 | | ± | 0.03 | *a* | 2.43E-02 | 2,3-Heptanedione |  |  |
| 131.107 | 131.1068 | C_7_H_14_O_2_H^+^ | 0.90 | ± | 0.25 | *a* | | 0.31 | ± | 0.13 | *b* | 1.06 | | ± | 0.40 | *a* | | 0.23 | ± | 0.06 | *b* | 1.41E-11 | 0.62 | ± | 0.44 | *a* | 0.80 | | ± | 0.26 | *a* | 2.62E-01 | Isoamyl acetate*; Butanoic acid 2-methyl ethyl ester*; Ethyl isovalerate* | [3] |  |
| 133.119 | 133.1178 | C_7_H_16_O_2_H^+^ | 0.07 | ± | 0.02 | *a* | | 0.02 | ± | 0.01 | *b* | 0.07 | | ± | 0.02 | *a* | | 0.03 | ± | 0.01 | *b* | 1.41E-12 | 0.05 | ± | 0.01 | *a* | 0.08 | | ± | 0.04 | *a* | 4.16E-02 |  |  |  |
| 135.138 | n.a. | unidentified | 1.47 | ± | 0.26 | *b* | | 0.26 | ± | 0.10 | *c* | 1.81 | | ± | 0.26 | *a* | | 0.29 | ± | 0.09 | *c* | 8.06E-26 | 1.90 | ± | 0.25 | *b* | 3.34 | | ± | 0.46 | *a* | 5.03E-08 |  |  |  |
| 143.107 | 143.1067 | C_8_H_14_O_2_H^+^ | 0.03 | ± | 0.01 | *a* | | 0.03 | ± | 0.01 | *a* | 0.04 | | ± | 0.01 | *a* | | 0.03 | ± | 0.01 | *a* | 2.17E-01 | 0.03 | ± | 0.01 | *b* | 0.08 | | ± | 0.02 | *a* | 7.36E-07 |  |  |  |
| 145.124 | 145.1223 | C_8_H_16_O_2_H^+^ | 0.09 | ± | 0.02 | *a* | | 0.05 | ± | 0.02 | *a* | 0.09 | | ± | 0.03 | *a* | | 0.05 | ± | 0.02 | *a* | 5.95E-05 | 0.07 | ± | 0.02 | *b* | 0.17 | | ± | 0.05 | *a* | 4.84E-06 | Isoamyl propionate* | [2] |  |
| 173.156 | 173.1536 | C_10_H_20_O_2_H^+^ | 0.02 | ± | 0.01 | *a* | | 0.01 | ± | 0.01 | *a* | 0.02 | | ± | 0.01 | *a* | | 0.01 | ± | 0.01 | *a* | 3.66E-01 | 0.02 | ± | 0.01 | *a* | 0.03 | | ± | 0.01 | *a* | 1.05E-02 | Ethyl octanoate | [1] |  |

**References**

1. Alves Z, Melo A, Figueiredo AR, Coimbra MA, Gomes AC, Rocha SM. 2015. Exploring the Saccharomyces cerevisiae volatile metabolome: indigenous versus commercial strains. *PLoS One* **10**:e0143641.
2. Barbosa C, Falco V, Mendes-Faia A, Mendes-Ferreira A. 2009. Nitrogen addition influences formation of aroma compounds, volatile acidity and ethanol in nitrogen deficient media fermented by Saccharomyces cerevisiae wine strains. *Biotechnol. Bioeng.* **108:**99-104
3. Casalone E, Fia G, Barberio C, Cavalieri D, Turbanti L, Polsinelli M. 1997. Genetic and biochemical characterization of Saccharomyces cerevisiae mutants resistant to trifluoroleucine. *Res. Microbiol.* **148**:613–623.
4. Cavalieri D, Townsend J, Hartl D. 2000. Manifold anomalies in gene expression in a vineyard isolate of Saccharomyces cerevisiae revealed by DNA microarray analysis. *Proc. Natl. Acad. Sci. U. S. A.* **97**:12369–12374. http://www.hubmed.org/display.cgi?uids=11035792.
5. Johansson HAB. 2011. Ionization and Fragmentation of Complex Molecules and Clusters. Stockholm Universitetsservice US-AB, 168 p.
6. Makhoul S, Romano A, Cappellin L, Spano G, Capozzi V, Benozzi E, Märk TD, Aprea E, Gasperi F, El-Nakat H, Guzzo J, Biasioli F. 2014. Proton-transfer-reaction mass spectrometry for the study of the production of volatile compounds by bakery yeast starters. *J. Mass Spectrom.* **49**:850–9.
7. Ugliano M, Henschke PA. 2009. Yeasts and Wine Flavour. In: Wine Chemistry and Biochemistry, New York: Springer, pp 313-392.
